# The effects of dietary patterns and food groups on symptomatic osteoarthritis: A systematic review

**DOI:** 10.1111/1747-0080.12781

**Published:** 2022-10-24

**Authors:** Jiayu Zeng, Daniella Kate Franklin, Arpita Das, Vasant Hirani

**Affiliations:** ^1^ Discipline of Nutrition and Dietetics School of Nursing and Midwifery, Charles Perkins Centre, The University of Sydney Sydney New South Wales Australia

**Keywords:** diet quality, dietary pattern, healthy diet, middle aged, osteoarthritis

## Abstract

**Aim:**

To systematically review current literature to determine the association between symptomatic osteoarthritis and dietary patterns, diet quality and food groups in adults aged ≥45 years.

**Methods:**

The review was registered on PROSPERO (CRD42021270891). Cochrane Central Library, Cumulative Index of Nursing and Allied Health Literature, Embase, Medline and Web of Science databases were searched. A total of 3816 records were identified. Eligible articles involved populations aged ≥45 years with symptomatic osteoarthritis, assessing dietary patterns, diet quality or food groups, with pain in joints as outcomes. The Joanna Briggs Institute Critical Appraisal Checklists were used for quality assessment. Grading of Recommendations, Assessment, Development and Evaluation was used to assess the certainty of evidence.

**Results:**

Six cohort studies were included. The Prudent dietary pattern and the Mediterranean dietary pattern reduced the progression of osteoarthritis symptoms. The Western dietary pattern increased symptomatic osteoarthritis progression. Increased total fibre consumption reduced symptomatic osteoarthritis progression and pain worsening, but the effects of fibre from each food group were inconclusive. Diet with high inflammatory potential increased risk of new onset symptomatic osteoarthritis, but the effects of overall diet quality were inconclusive.

**Conclusions:**

The Prudent dietary pattern showed the highest protection on symptomatic osteoarthritis in adults aged 45 years and over. The body of evidence is limited, suggesting that further research is needed to corroborate the estimated effect at a high certainty of evidence, and to incorporate previously unstudied dietary patterns and food groups. Identifying the most beneficial dietary pattern may inform future guidelines for reducing symptomatic osteoarthritis in middle aged and older adults.

## 1 INTRODUCTION

Osteoarthritis is a chronic and progressive degenerative joint disease, leading to a gradual health and physical function decline.^1^ It is the most common form of arthritis leading to knee and hip replacements in Australia, estimated at 9.3% of the total population.^2^ The aetiology is categorised by bone joint cartilage deterioration, subchondral bone remodelling, synovial inflammation and articular cartilage loss.^3^ There are radiographic and clinical definitions of osteoarthritis. Definitions of radiographic osteoarthritis differ among studies. For example, radiographic osteoarthritis can be defined as a Kellgren–Lawrence grade of 4 at baseline,^4^ whilst another study^5^ defines radiographic osteoarthritis as a Kellgren–Lawrence grade of ≥2 at follow up. The inflammatory response and the reduction of smooth movements caused by the physiological changes leads to clinical symptoms of pain, aching or stiffness.^6^, ^7^ However, not all individuals with joint symptoms are diagnosed with radiographic osteoarthritis.^8^ The presence of such clinical symptoms plus the radiographic osteoarthritis is named as symptomatic osteoarthritis.^8^ The activities of daily living and the quality of life are largely impacted in individuals with osteoarthritis.^9^ Therefore, osteoarthritis is considered one of the leading causes of disability among the older population.^10^

The prevalence of osteoarthritis is strongly related to age and gender, increasing significantly from 45–54 years to 55–64 years (9.7%–20.7%) according to the Australian National Health Survey 2017–2018.^2^ It affects approximately 6% of Australian males (*n* = 805 800, estimated) and 10% of Australian females (*n* = 749 200, estimated).^2^ Osteoarthritis accounted for 19% of the total burden of disease due to musculoskeletal conditions in Australia in 2015.^11^ Osteoarthritis' burden is projected to increase exponentially due to Australia's ageing and obese population, with prevalence expected to reach 3 million Australians by 2032^1^ and 130 million internationally by 2050.^12^

There are multiple risk factors involved in the generation and progression of osteoarthritis. Unmodifiable risk factors include age, gender, ethnicity, genetics, joint malalignment, and family history.^6^, ^8^, ^9^ Congenital or acquired joint shape and malalignment are associated with greater osteoarthritis risk in younger individuals.^13^, ^14^ Modifiable risk factors include diet, overweight and obesity, injury, occupational overuse of joints, physical activity, bone density, joint laxity, and muscular weakness.^6^, ^8^, ^9^ The management of osteoarthritis involves education, physical therapy, diet and exercise interventions, weight loss and surgery.^6^ The use of medications reduces pain, such as paracetamol, or reduces swelling and pain, such as the use of non‐steroidal anti‐inflammatory drugs, including diclofenac sodium, celecoxib, meloxicam and naproxen.^6^

As both a risk factor and a management strategy, the association between diet and osteoarthritis has been a focused topic. Low intake of certain nutrients, for instance Vitamin D, Vitamin C, Vitamin E, Vitamin K and magnesium, have been found to be associated with increasing the risk of osteoarthritis progression or worsening of symptoms.^15^, ^16^, ^17^, ^18^, ^19^ In contrast, randomised controlled trials of supplementing single nutrients Vitamin D, E and K have not shown any protective effects for osteoarthritis.^20^, ^21^, ^22^ This suggests that focusing on individual nutrients may be insufficient, and the effects of food groups and overall dietary patterns should be the focus when studying diet‐disease relationships.^23^, ^24^

Other than diet, overweight and obesity is another osteoarthritis risk factor. It increases weight‐bearing joint and cartilage load and contributes to degradation.^25^, ^26^ Approximately 70% of osteoarthritis is preventable by avoiding excess weight gain and joint injuries.^1^ Foods have synergistic health and disease effects,^27^ influencing clinical nutritional recommendations on osteoarthritis.^28^, ^29^ There are limited primary studies, such as randomised controlled trials and cohort studies, and no systematic reviews that have shown that the relative risk of osteoarthritis and presence of symptomatic osteoarthritis is negatively associated with anti‐inflammatory diets (e.g., DASH, Prudent and Mediterranean diet),^24^, ^30^, ^31^, ^32^, ^33^ and positively associated with pro‐inflammatory diets (e.g., Western diet).^4^, ^32^, ^34^ However, associations between diet and osteoarthritis‐specific symptoms after follow‐up remains inconclusive, and no systematic analysis has been conducted to date to assess the quality of the evidence. As prevalence increases in individuals 45+ years with greater progression with age, this review aims to scope associations of dietary patterns, diet quality, and food groups with symptomatic osteoarthritis in adults aged 45 years and older with joint pain, aching or stiffness, with radiographic osteoarthritis. This systematic review will synthesise available evidence in the literature on associations of the consumption of pro‐ and anti‐inflammatory diets and presence of symptomatic osteoarthritis, using outcome measurements such as the Western Ontario and McMaster Universities Osteoarthritis Index (WOMAC) pain scale. This will subsequently add to the body of evidence in the literature.

## 2 METHOD

This systematic review was registered at the PROSPERO International Prospective Register of Systematic Reviews prior to the study commencement (Registration number: CRD42021270891). The review was reported by following the PRISMA (Preferred Reporting Items for Systematic Reviews and Meta‐Analyses) guidelines (see full PRISMA checklist in Table S1).

A comprehensive literature search was conducted on 2nd September, 2021 using electronic databases Cochrane CENTRAL Library (via Ovid), Cumulative Index of Nursing and Allied Health Literature (CINAHL), Embase (via Ovid), MEDLINE (via Ovid) and Web of Science (Core Collection). Reference lists of final included articles were searched by 4th October, 2021 subsequent to database search. Three groups of key terms indicating the population, exposure and outcome of interest were adapted from an original search strategy to each of the databases. The original search strategy was formed as follows (with full search strategies in Table S2).

For extensive and thorough research on dietary patterns and symptomatic osteoarthritis, the search strategy included the terms ‘diet’, OR ‘diet therapy’, OR ‘diet pattern’, OR ‘diet intake’, OR ‘diet treatment’, OR ‘diet restrict’, OR ‘diet therapy’, OR ‘meal pattern’, OR ‘eating pattern’, OR ‘food pattern’ OR ‘eating pattern’ OR ‘food pattern’ OR ‘diet habit’ OR ‘food’ OR ‘food group’ AND ‘Symptomatic Diseases’ OR ‘symptomatic’ AND ‘osteoarthritis’ OR ‘Cartilage’ OR ‘chondral’ OR ‘meniscal’ OR ‘meniscus’ OR ‘bone marrow’ OR ‘subchondral’ OR ‘osteophyte’ OR ‘effusion’ OR ‘synovitis’ OR ‘ligament’ OR ‘attrition’ OR ‘fat pad’ AND ‘Joint’ AND ‘pain’, OR ‘aching’ OR ‘stiffness’, OR ‘tightness’ AND ‘middle aged’, OR ‘45+ years’, OR ‘older’ OR ‘ageing’, OR ‘aged’, OR ‘elder’, OR ‘elderly’.

The first and second screenings were conducted in duplicate by two reviewers. The first screening included assessing the titles and abstracts of each study, and the second screening included a full‐text review. Both screenings were against the inclusion and exclusion criteria listed in Table 1. Eligible articles involved populations aged ≥45 years with symptomatic osteoarthritis, assessing dietary patterns, diet quality, or food groups, with pain, stiffness in joints and physical function as outcomes. Eligible study designs were randomised controlled trials and cohort studies. Consensus was reached after comparing and discussing the results of screening by the two reviewers, and another two reviewers were available if consensus could not be reached by two reviewers.

**TABLE 1:** PICOS inclusion/exclusion criteria

Parameter	Inclusion criteria	Exclusion criteria
Population	Individuals aged 45 years and older with pain, aching or stiffness in a joint with radiographic osteoarthritis.^2^	Individuals aged <45 years with major illnesses. Individuals who underwent surgical treatment for osteoarthritis and individuals with neurological or cardiovascular diseases. Individuals in hospitals or institutions.
Intervention or Exposure	Interventions of dietary patterns, diet quality or food groups including all modes of deliveries such as direct meal deliveries and dietary advice provided by trained professionals. Interventions involving dietary patterns, diet quality or food groups with the manipulation of nutrient composition with a whole diet approach or dietary patterns supplemented with food items. Exposures to dietary patterns, diet quality or food groups assessed by one or more of the following methods: dietary history taken by a trained professional, food frequency questionnaire, 24‐h recall or weighed food record.	Dietary patterns supplemented with supplements Studies including other interventions in addition to dietary patterns. Non‐food exposures involving single nutrients or supplements. Exposures to dietary patterns assessed indirectly through grocery item lists.
Comparison	Inactive control diet (such as a placebo, no treatment, usual care without dietary advice, or a waiting list control). Comparator diet. Non‐exposure to the diet.	Not applicable.
Outcomes	The primary outcomes included pain, stiffness in a joint and physical function.	Studies that did not assess symptoms that are specific to osteoarthritis. For example, knee structure changes, radiographic osteoarthritis progression, quality of life, and so on.
Study design	Randomised controlled trials, cluster randomised controlled trials, pseudo‐randomised controlled trial, non‐randomised controlled clinical trials, cluster trials, prospective cohort studies, retrospective cohort studies.	Controlled before‐after studies, interrupted time studies without a control group, cross‐sectional studies, case series, case reports, non‐study based sources, narrative reviews and systematic reviews. Interrupted time‐series studies with a control group, case–control studies, cross‐sectional studies and nested case–control studies.
Language	English	Other than English

Data were extracted in duplicate from each study by two reviewers and the extracted data were checked by two reviewers. Data extracted included study details (author, year of publication, study's country, study design, setting, recruitment, eligibility criteria, follow‐up duration), population characteristics (age, sex, race, sample size, withdrawal or exclusions, underlying disease status of participants), intervention or exposure (dietary pattern studied, diet assessment method, level of dietary control, randomisation and comparator) and outcomes, statistical method and potential confounders. Authors were contacted for any missing full text or data.

The quality assessment of included studies was conducted in duplicate by two reviewers. The Joanna Briggs Institute Critical Appraisal Checklist was used for risk of bias assessment at the cohort study level.^36^ The Joanna Briggs Institute appraisal tool consists of 11 questions with 4 answer options: ‘yes’, ‘no’, ‘unclear’ and ‘not applicable’ (Table 4). The final judgement of including or excluding certain studies was determined by the overall appraisal. Studies which answered more than three ‘no’ or ‘unclear’ were considered poor quality and therefore were excluded. Any disagreement was discussed by two reviewers, and two additional reviewers involved if consensus was not reached.

The certainty of the body of evidence for each outcome as effect estimates by dietary pattern was assessed using GRADE (Grading of Recommendations Assessment, Development and Evaluation) and was categorised as ‘high’, ‘moderate’, ‘low’ and ‘very low’. The certainty of evidence could be downgraded either one or two levels based on five factors: risk of bias, inconsistency, imprecision, indirectness and publication bias. ‘Serious’ indicates one level downgrading and ‘very serious’ indicates two levels downgrading. GRADE guideline 6 suggests population sample sizes over 400 are likely to meet optimal information size.^40^ The downgrading of imprecision was determined by if the study had a small sample size <400, and/or wide 95% confidence intervals (CIs). Studies were downgraded by one level if CIs included both null effect and appreciable benefit (CI < 0.75) or harm (CI > 1.25). Studies were downgraded by two levels if CIs included both appreciable benefit and harm. Consensus of the GRADE results was reached between the two reviewers, and a third reviewer was available if consensus could not be reached by two reviewers.

## 3 RESULTS

**FIGURE 1:**
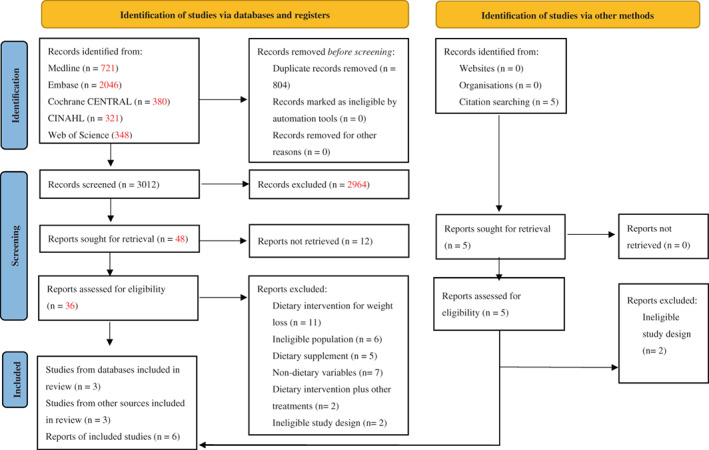
Flow diagram of the literature search. CENTRAL, Cochrane Central Register of Controlled Trials; CINAHL, Cumulative Index of Nursing and Allied Health Literature.

There were 3816 records identified from the database on 2nd September 2021. After removing 804 duplicates, 3012 papers were screened by title and abstract against the inclusion and exclusion criteria. From these, 48 articles were retrieved in full‐text to assess eligibility. After failed attempts to contact the authors, 12 papers failed to be assessed for full‐text screening. Besides these, reasons for exclusion included dietary intervention for weight loss involving fasting or energy restriction (*n* = 12), ineligible population (*n* = 6), dietary intervention involving supplements (*n* = 5), non‐dietary variables (*n* = 7), exposure involving diet and other treatments spontaneously (*n* = 2), and ineligible study design (*n* = 2). Three articles were included in the review,^4^, ^37^, ^39^ and further records were identified from reference lists of eligible articles (*n* = 5) by 4th October 2021. Of these, two articles were removed due to ineligible study design.^30^, ^41^ A total of six articles were included for data extraction and quality assessment. The detail of study selection is presented in the PRISMA flow diagram (Figure 1).

The characteristics of included articles are presented in Table 2. Studies involved participants with both males and females, aged 45 years and over and with a history of osteoarthritis symptoms (*n* = 6). All six articles were prospective cohort studies from the United States (*n* = 4),^4^, ^34^, ^37^, ^38^ Australia (*n* = 1),^39^ and the United Kingdom (*n* = 1).^5^ Of these, four articles collected data from the same cohort study, the Osteoarthritis Initiative,^4^, ^34^, ^37^, ^38^ one article used the data from the Vitamin D Effect on Osteoarthritis (VIDEO) study^39^ and one article compared the data from Osteoarthritis Initiative and the Framingham study.^5^

**TABLE 2:** Characteristics of included studies

Study details	Sample size and characteristics	Recruitment and intervention time	Inclusion/exclusion criteria	Outcome measure	Dietary intake measurements
*American OAI Cohort*					
Dai et al.^37^ the United States, prospective cohort, 96 months	*Final sample*: 4470/4796 (93.2% participation) *Withdrew/Excluded*: 326 3703 knees with OA (Ages 45–79 years) 41.5% men	American OAI Launched by the NIH, from February, 2004 to May 2006	*Inclusion*: Participants Aged 45 and over, overweight, previous knee injury or surgery, knee pain during the past year. Family history of knee replacement. *Exclusion*: Rheumatoid arthritis, joint replacements in both knees, unable to walk without assistance, unable to undergo MRI of the knee, history of TKR or PKR at baseline.	*Methods/Measures*: Examiners studied knee pain development patterns over 96 months using the WOMAC pain subscale of five activity items: walking, stair climbing, nocturnal, rest and weight bearing.	*Methods/Measures*: Validated Block Brief 2000 FFQ at baseline, calculated based on the food composition database for nutrients in the SNHNES and separated into quartiles of fibre intake: (I) Total energy (kcal/day), median IQR, (II) total dietary fibre (g/day), median IQR, (III) grain fibre (g/day), median IQR, (IV) fruit and vegetable fibre (g/day), median IQR and (V) nut and legume fibre (g/day) median IQR).
Veronese et al.^38^ the United States, prospective cohort, 48 months	*Final sample*: 4330/4796 (90.2% participation). *Withdrew/excluded*: 466 (63 = TKR, 118 = insufficient information and 285 = no data regarding outcomes of interest). (Ages 45–79 years) 58.0% females and 42% males at baseline. Mean age: 61.1 years	American OAI recruited participants across four American states between February 2004 and May 2006.	*Inclusion*: Participants aged between 45 and 79 years of age from OAI, validated exposure (Mediterranean Diet adherence) and outcomes (pain worsening, SxOA and/or ROA). *Exclusion*: Participants had total knee replacement at baseline.	*Methods/Measures*: Question regarding knee pain: ‘During the past 30 days, have you had pain, aching or stiffness in your right/left knee on most days?’ Knee pain: WOMAC (Western Ontario and McMaster Universities Osteo‐arthritis Index) pain subscale.	*Methods*: Block Brief 2000 food frequency (FFQ). *Measures*: Adherence to a Mediterranean diet presented by aMED.
Liu et al.^34^ the United States, longitudinal prospective cohort study, 48 months	*Final sample*: 2940/4796 *Withdrew/excluded*: 1856/4796 (61.3% participation). (Ages 47–79 years) 58.5% female; mean BMI at baseline 28.0 kg/m^2^.	The American OAI recruited participants from four US states over a 48 month follow up.	*Inclusion*: Participants were aged between 47 and 79 years of age from OAI, with realistic energy intake, with incident SxOA or ROA. *Exclusion*: Participants with total knee replacement at baseline, with missing dietary data, with extreme calories intake, with missing SxOA or ROA data at baseline, with prevalent SxOA or ROA at baseline, with missing incident SxOA or ROA data at 48‐month follow‐up.	*Methods/Measures*: SxOA: pain, aching or stiffness on more than 15 days of a month during the past year.	*Methods*: Block Brief 2000 Food Frequency Questionnaire *Measures*: Habitual dietary intake of nutrients and foods at baseline to estimate dietary inflammatory potential presented by quartiles of DII score per 1000 kcal energy
Xu et al.^4^ the United States, prospective cohort, 72 months.	*Final sample*: 2757/4134 (66.7% participation) *Number of loss of follow‐up*: 129 in year 2, 139 in year 3, 221 in year 4 and 1377 in year 6 (due to death, TKR or nonresponse) (Ages 45–79 years) 40.5% male; Mean age: 62.1 ± 9.0; 78.8% white, 18.2% African American, 3.1% others; 16.7% high school education or lower, 46.1% college education, 37.1% higher than college education; 13.2% ≤ $25 K family income, 25.6% $25–50 K family income, 34.6% $50–100 K family income, 20.5% > $100 K family income, 8.9% depressed; 53.4% non‐smoker, 6.2% smoker, 40.4% ex‐smoker; 18.2% BMI <25, 39.9% BMI 25–30, 41.9% BMI ≥30	American OAI launched by the NIH, from February, 2004 to May 2006	*Inclusion*: Participants aged 45 and over from OAI with mild to moderate KOA in at least 1 knee (KL grade of 1, 2 or 3) at baseline based on x‐ray reading. *Exclusion*: Participants without OA, with severe ROA (KL grade of 4 at baseline), with lateral joint space narrowing, with unrealistic total daily calorie intake (<800 or >4200 kcal for men, <500 or >3500 kcal for women).	*Measures/Methods*: SxOA: Validated WOMAC pain, functional disability and total scores.	*Methods*: Validated 70‐item Block Brief FFQ at baseline. *Measures*: Average frequency of consumption for each food item aggregated into 25 food groups. Diet quality which is represented by the scores of Western and Prudent diet separately and a combined score derived by factor‐loading matrix using Block Brief FFQ.
*American OAI and Framingham Offspring Cohorts*					
Dai et al.^5^ the United States, the United Kingdom, prospective cohort, 2 years for OAI and 9.5 years for the Framingham study.	*Final sample*: OAI: 4256/4796 Framingham Study: 1137/1268 *Withdrew/excluded*: OAI: 540 (89% participation). Framingham Study: 131 (88.1%) participation. (Ages 45–79 years) OAI: 41.7% men; mean age 61.4 years; BMI 28.6%. Framingham Study: 45.5% men; mean age 53.9 years; BMI 27	The American OAI recruited participants from 2004 to 2006 in the United States. Participants were also recruited from the Framingham Offspring cohort assembled in 1971.	*Inclusion*: Participants who were recruited were aged between 45 and 79 years of age, from OAI with incident ROA, SxOA, or pain worsening, or from the Framingham study, with incident ROA or SxOA and valid fibre and energy intake. *Exclusion*: OAI: loss to follow‐up at 48 months, with extreme calories intake, with total knee replacement at baseline, with missing or prevalent SxOA or ROA at baseline, with missing WOMAC pain score at baseline or at 48 months. Framingham: Loss to follow‐up at exam 7, with missing data on fibre intake.	*OAI/Framingham Study* *Methods/Measures*: SxOA: response to the question ‘During the past 30 days, have you had pain, aching, or stiffness in your right/left knee on most days? And ≥14% in WOMAC pain score difference between baseline and each follow‐up.	*OAI* *Methods*: Block Brief 2000 food frequency (FFQ) *Measures*: Dietary fibre intake including total dietary fibre, cereal fibre, fruit and vegetable fibre and nut and legume fibre. *Framingham Study*: *Methods*: Harvard validated *Measures*: Habitual dietary intake and dietary fibre intake
*VIDEO Study*					
Ruan et al.^39^ Australia, prospective cohort, 24 months	*Final sample*: 392/413 (94.9% participation) *Withdrew/Excluded*: 21 Mean age: 63.26 years (Ages 50–79 years) 50.51% male Mean height: 168.36 cm Mean weight: 83.87 kg Mean BMI: 29.56 kg/m^2^	The VIDEO study, conducted between June 2010 and December 2013. Recruitment began from June 2010 to December 2011 in Tasmania and Victoria, Australia	*Inclusion*: Aged between 50 and 79 years, had clinical knee OA compiled with the ACR criteria, pain score >20 mm on visual analog scale, had an ACR function class rating of I, II and III, good health scored 0–2 on a 5 point Likert scale (from 0 indicating very good health to 4 indicating very poor health). *Exclusion*: Grade 3 knee ROA, contraindication to magnetic resonance imaging (MRI), rheumatic diseases and other severe diseases.	*Methods/Measures*: Assessment of OA symptoms at baseline, 3, 6, 12 and 24 months using WOMAC OA Index.	*Methods/Measures*: (i) Dietary intake was assessed at baseline using the DQES v2. Participants reported usual consumption over the past 12 months of 74 foods on a 10‐point frequency scale. (ii) Diet quality was assessed using the ARFS calculated based on DQES v2 items.

Abbreviations: aMED, Mediterranean Diet Score; ACR, American College of Rheumatology; AQoL‐4D, four‐dimensional assessment of quality of life; ARFS, Australian Recommended Food Score; BMI, Body Mass Index; BMLs, bone marrow lesions; DII, Dietary Inflammatory Index; DQES v2, Dietary Questionnaire for Epidemiological Studies version 2; FFQ, Food Frequency Questionnaire; g/day, grams per day; IQR, interquartile range; kcal/day, kilocalories per day; JSW, joint space width; K, $1000; KL, Kellgren–Lawrence; KOA, knee osteoarthritis; MRI, magnetic resonance imaging; NIH, National Institutes of Health; OA, osteoarthritis; OAI, American Osteoarthritis Initiative; PHQ‐9, 9‐Items Patient Health Questionnaire; PKR, partial knee replacement; QoL, quality of life; ROA, radiographic osteoarthritis; SNHNES, Second National Health and Nutrition Examination Survey; SxOA, symptomatic osteoarthritis; TKR, total knee replacement; VIDEO, Vitamin D Effect on Osteoarthritis; WOMAC, Western Ontario and McMaster Universities Osteoarthritis Index.

All six studies included adequate follow‐up periods, ranging from 2 to 9.5 years, and sufficient sample size, ranging from 392 to 4470 participants (*n* = 6). One study had greater than 30% loss to follow‐up, over a 72 month period.^4^ However, statistical analysis was applied for adjusting follow‐up time points, which was considered acceptable (Table 4). One study had greater than 30% exclusion rate due to ineligible data (participants with prior total knee replacement at baseline (*n* = 64), participants with missing dietary data (*n* = 129), participants with extreme energy intake (*n* = 111), participants with missing symptomatic osteoarthritis data at baseline (*n* = 168), participants with prevalent symptomatic osteoarthritis at baseline (*n* = 1246), and participants with missing incident symptomatic osteoarthritis data at 48 month follow‐up (*n* = 138)).^34^ Participants with major diseases were excluded from all six articles, and participants with severe osteoarthritis were excluded from two articles.^4^, ^39^ In addition, all studies utilised validated methods to measure exposure and outcome. Dietary exposures included Western pattern and Prudent pattern measured by 70‐item Block Brief food frequency questionnaire^4^; Mediterranean diet,^38^ inflammatory potential of diet,^34^ and dietary fibre intake^5^, ^37^ measured by Block Brief 2000 FFQ; and overall diet quality measured by Dietary Questionnaire for Epidemiological Studies v2^39^ and 70‐item Block Brief food frequency questionnaire.^4^ The majority of studies only measured exposure once at baseline except the Framingham study, which measured dietary intake at baseline and 4 years later.^5^ In terms of outcomes, two studies had osteoarthritis symptom progression,^4^, ^39^ two studies had incident symptomatic osteoarthritis,^5^, ^34^ one study had pain progression^37^ and one study had both incident symptomatic osteoarthritis and pain progression.^38^ All studies measured outcome several times at each follow‐up time point (Table 2 column 1).

The quality of evidence for each outcome was presented in Table 3. All six articles had less than three questions which answered ‘no’ or ‘unclear’ using Joanna Briggs Institute Critical Appraisal Checklists (Table 4). More details of individual articles are discussed below.

**TABLE 3:** Statistical analyses of cohort studies

Outcome	Reference (Year), study design, covariates in fully adjusted model	Statistical analyses (methods and confounders)	Outcome result
*Overall diet quality*			
SxOA progression	Xu et al. (2020)^4^	*Dietary patterns and KL progression*: Separate Cox proportional hazards models with discrete likelihood method for follow‐up time. *Dietary patterns and JSW/Dietary patterns and symptomatic KOA progression*: Linear mixed models. *Confounders*: age, sex, race, baseline KL grade, assessed depression (defined as the CES‐D 20 items scale >16), BMI, weight change from baseline, physical activity, total energy intake, traumatic knee injury, knee surgery, income, education, smoking and alcohol intake and pain relief medication usage.	*Quartiles of the combined diet score, more healthy diet: OR (95% CI), p‐value, cases/person‐ years* Q1: 1.00, referent, 591/1128 Q2: 1.19 (1.01, 1.41), *p* = 0.04, 617/1176 Q3: 1.22 (1.03, 1.44), *p* = 0.02, 613/1114 Q4: 1.29 (1.08, 1.53), *p* < 0.01, 619/1044 *p*‐trend <0.01 (significant)
Knee pain VAS	Ruan et al. (2021)^39^	Associations of diet quality with OA symptoms, knee symptoms, QoL and OA comorbid conditions estimated using the mixed‐effects linear regression model. *Confounders*: Age, sex, BMI, serum vitamin D level, energy intake, visit time, education, work status and work type.	*β* (95% CI): −0.12 (−0.38, 0.14); *p* = 0.36 (non‐significant)
WOMAC joint stiffness			*β* (95% CI): −0.28 (−0.77, 0.20); *p* = 0.25 (non‐significant)
WOMAC knee pain			*β* (95% CI): −0.55 (−1.52, 0.42); *p* = 0.27 (non‐significant)
Total WOMAC			*β* (95% CI): −4.00 (−8.91, 0.90); *p* = 0.11 (non‐significant)
WOMAC physical dysfunction			*β* (95% CI): −3.14 (−6.75, 0.48); *p* = 0.09 (non‐significant)
*Dietary Inflammatory Potential*			
SxOA	Liu et al. (2020)^34^	Test for linear trend using the median value of each quartile of E‐DII score as a continuous variable in the regression model. Alternative analyses were conducted fitting E‐DII as a continuous variable in models. *Confounders*: Age, sex, race, educational attainment, annual income, physical activity and tobacco use.	*Quartiles of E‐DII score: OR (95% CI), no. of cases* Q1: 1.00 referent, 218/1472 Q2: 1.13 (0.92, 1.40), 237/1470 Q3: 1.27 (1.04, 1.56), 260/1470 Q4: 1.43 (1.16, 1.76), 263/1468 *p* = 0.001 (significant)
*Western diet*			
SxOA progression	Xu et al. (2020)^4^	*Dietary patterns and KL progression*: Separate Cox proportional hazards models with discrete likelihood method for follow‐up time. *Dietary patterns and JSW/Dietary patterns and symptomatic KOA progression*: Linear mixed models. *Confounders*: age, sex, race, baseline KL grade, assessed depression (defined as the CES‐D 20 items scale >16), BMI, weight change from baseline, physical activity, total energy intake, traumatic knee injury, knee surgery, income, education, smoking and alcohol intake and pain relief medication usage.	*Quartiles of the Western diet score: OR (95% CI), p‐value, cases/person‐ years* Q1: 1.00 referent, 594/1156 Q2: 1.06 (0.89, 1.25), *p* = 0.51, 609/1130 Q3: 1.15 (0.97, 1.37), *p* = 0.11, 608/1105 Q4: 1.26 (1.06, 1.50), *p* < 0.01, 629/1071 *p*‐trend <0.01 (significant)
*Prudent diet*			
SxOA progression	Xu et al. (2020)^4^	*Dietary patterns and KL progression*: Separate Cox proportional hazards models with discrete likelihood method for follow‐up time. *Dietary patterns and JSW/Dietary patterns and symptomatic KOA progression*: Linear mixed models. *Confounders*: age, sex, race, baseline KL grade, assessed depression (defined as the CES‐D 20 items scale >16), BMI, weight change from baseline, physical activity, total energy intake, traumatic knee injury, knee surgery, income, education, smoking, and alcohol intake and pain relief medication usage.	*Quartiles of the Prudent diet score: OR (95% CI), p‐value, cases/person‐years* Q1: 1.00 referent (lowest), 618/1077 Q2: 0.90 (0.76, 1.07), *p* = 0.22, 611/1094 Q3: 0.90 (0.76, 1.06), *p* = 0.21, 614/1141 Q4: 0.71 (0.60, 0.84), *p* < 0.01, 597/1150 *p*‐trend <0.01 (significant)
*Mediterranean diet*			
Pain worsening	Veronese et al. (2019)^38^	Multivariable Poisson regression analysis *p* values for trends: Jonckheeree Terpstra test for continuous variables, Mantel–Haenszel Chi‐square test for categorical variables. *Confounders*: Race; educational attainment; BMI; yearly income; depressive symptoms measured using CES‐D; smoking habits; physical activity level evaluated using PASE; Charlson Comorbidity Index score; daily energy intake; number of medications at baseline.	*Quintiles of aMED score: RR (95% CI), p‐value* aMED 24: 1.00 referent aMED 25–27: 0.98 (0.95, 1.02), *p* = 0.45 aMED 28–30: 0.99 (0.95, 1.03), *p* = 0.72 aMED 31–32: 0.99 (0.94, 1.03), *p* = 0.53 aMED >32: 0.96 (0.91, 0.999), *p* = 0.047 Increase in one SD: 0.98 (0.97, 0.998), *p* = 0.04 (significant)
Symptomatic knee OA	Veronese et al. (2019)^38^	Multivariable Poisson regression analysis *p* values for trends: Jonckheeree Terpstra test for continuous variables, Mantel–Haenszel Chi‐square test for categorical variables. *Confounders*: Race; educational attainment; BMI; yearly income; depressive symptoms measured using CES‐D; smoking habits; physical activity level evaluated using PASE; Charlson Comorbidity Index score; daily energy intake; number of medications at baseline.	*Quintiles of aMED score: RR (95% CI), p‐value* aMED ≤24: 1.00 referent aMED 25–27: 0.94 (0.85, 1.04), *p* = 0.25 aMED 28–30: 0.91 (0.82, 1.009), *p* = 0.07 aMED 31–32: 0.93 (0.83, 1.05), *p* = 0.26 aMED >32: 0.91 (0.82, 0.998), *p* = 0.048 Increase in one SD: 0.96 (0.93, 0.997), *p* = 0.04 (significant)
*Dietary fibre intake*			
WOMAC pain (mild)	Dai et al. (2017a)^37^	Group‐based trajectory modelling procedure using a multinomial modelling strategy. *Confounders*: demographics, tobacco and alcohol use, depressive symptoms measured by CES‐D, knee injury and surgery, medication use, and physical activity assessed by PASE.	*Quartiles of total fibre (median [IQR] g/day): OR(95% CI), no. of cases* Q1 (8.6 [6.3–11.3]): 1.00 referent, 378/3703 Q2 (12.5 [9.9–15.6]): 0.88 (0.66, 1.17), 374/3703 Q3 (15.2 [12.2–19.0]): 0.76 (0.58, 1.01), 346/3703 Q4 (20.6 [16.2–26.5]): 0.87 (0.65, 1.16), 369/3703 *p*‐trend = 0.28 (non‐significant)
			*Quartiles of cereal grain fibre (median [IQR] g/day): OR (95% CI), no. of cases* Q1 (3.7 [2.5–5.2]): 1.00 referent, 366/3703 Q2 (5.0 [3.5–6.8]): 1.28 (0.96, 1.69), 388/3703 Q3 (5.7 [3.9–8.0]): 1.00 (0.76, 1.31), 376/3703 Q4 (6.8 [4.5–9.8]): 1.01 (0.77, 1.33), 335/3703 *p*‐trend = 0.6 (non‐significant)
			*Quartiles of fruit and vegetable fibre (median [IQR] g/day): OR (95% CI), no. of cases* Q1 (3.8 [2.6–5.4]): 1.00 referent, 367/3703 Q2 (6.1 [4.5–8.1]): 1.13 (0.84, 1.51), 369/3703 Q3 (7.8 [6.0–10.0]): 1.17 (0.88, 1.56), 384/3703 Q4 (10.5 [7.8–14.0]): 0.90 (0.68, 1.20), 345/3703 *p*‐trend = 0.35 (non‐significant)
			*Quartiles of legume and nut fibre (median [IQR] g/day):OR (95% CI), no. of cases* Q1 (0.8 [0.4–1.4]): 1.00 referent, 380/3703 Q2 (1.4 [0.8–2.1]): 1.03 (0.78, 1.36), 382/3703 Q3 (1.6 [1.0–2.6]): 0.88 (0.67, 1.17), 334/3703 Q4 (2.3 [1.2–4.0]): 1.09 (0.83, 1.44), 371/3703 *p*‐trend = 0.57 (non‐significant)
WOMAC pain (moderate)			*Quartiles of total fibre (median [IQR] g/day): OR(95% CI), no. of cases* Q1 (8.6 [6.3–11.3]): 1.00 referent, 269/3703 Q2 (12.5 [9.9–15.6]): 0.76 (0.58, 1.02), 240/3703 Q3 (15.2 [12.2–19.0]): 0.70 (0.52, 0.94), 240/3703 Q4 (20.6 [16.2–26.5]): 0.57 (0.42, 0.77), 189/3703 *p*‐trend = 0.0004 (significant)
			*Quartiles of cereal grain fibre (median [IQR] g/day): OR (95% CI), no. of cases* Q1 (3.7 [2.5–5.2]): 1.00 referent, 257/3703 Q2 (5.0 [3.5–6.8]): 1.08 (0.80, 1.45), 242/3703 Q3 (5.7 [3.9–8.0]): 0.91 (0.68, 1.22), 230/3703 Q4 (6.8 [4.5–9.8]): 0.98 (0.73, 1.32), 227/3703 *p*‐trend = 0.59 (non‐significant)
			*Quartiles of fruit and vegetable fibre (median [IQR] g/day): OR (95% CI), no. of cases* Q1 (3.8 [2.6–5.4]): 1.00 referent, 268/3703 Q2 (6.1 [4.5–8.1]): 0.93 (0.69, 1.25), 243/3703 Q3 (7.8 [6.0–10.0]): 0.85 (0.63, 1.15), 230/3703 Q4 (10.5 [7.8–14.0]): 0.60 (0.45, 0.81), 199/3703 *p*‐trend = 0.0004 (significant)
			*Quartiles of legume and nut fibre (median [IQR] g/day):OR (95% CI), no. of cases* Q1 (0.8 [0.4–1.4]): 1.00 referent 245/3703 Q2 (1.4 [0.8–2.1]): 0.98 (0.73, 1.32), 242/3703 Q3 (1.6 [1.0–2.6]): 0.92 (0.69, 1.23), 245/3703 Q4 (2.3 [1.2–4.0]): 0.77 (0.57, 1.05), 208/3703 *p*‐trend = 0.09 (non‐significant)
WOMAC pain (severe)			*Quartiles of total fibre (median [IQR] g/day): OR(95% CI), no. of cases* Q1 (8.6 [6.3–11.3]): 1.00 referent, 108/3703 Q2 (12.5 [9.9–15.6]): 0.59 (0.38, 0.90), 67/3703 Q3 (15.2 [12.2–19.0]): 0.61 (0.39, 0.93), 70/3703 Q4 (20.6 [16.2–26.5]): 0.41 (0.24, 0.68), 53/3703^a^ *p*‐trend = 0.0006 (significant)
			*Quartiles of cereal grain fibre (median [IQR] g/day): OR (95% CI), no. of cases* Q1 (3.7 [2.5–5.2]): 1.00 referent, 102/3703 Q2 (5.0 [3.5–6.8]): 1.04 (0.69, 1.57), 84/3703 Q3 (5.7 [3.9–8.0]): 0.65 (0.41, 1.03), 64/3703 Q4 (6.8 [4.5–9.8]): 0.55 (0.33, 0.91), 48/3703 *p*‐trend = 0.006 (significant)
			*Quartiles of fruit and vegetable fibre (median [IQR] g/day): OR (95% CI), no. of cases* Q1 (3.8 [2.6–5.4]): 1.00 referent, 94/3703 Q2 (6.1 [4.5–8.1]): 0.89 (0.57, 1.39), 72/3703 Q3 (7.8 [6.0–10.0]): 0.85 (0.55, 1.33), 67/3703 Q4 (10.5 [7.8–14.0]): 0.61 (0.39, 0.95), 67/3703 *p*‐trend = 0.02 (significant)
			*Quartiles of legume and nut fibre (median [IQR] g/day):OR (95% CI), no. of cases* Q1 (0.8 [0.4–1.4]): 1.00 referent, 104/3703 Q2 (1.4 [0.8–2.1]): 0.60 (0.38, 0.94), 63/3703 Q3 (1.6 [1.0–2.6]): 0.70 (0.46, 1.09), 73/3703 Q4 (2.3 [1.2–4.0]): 0.69 (0.44, 1.09), 56/3703 *p*‐trend = 0.23 (non‐significant)
Pain worsening	Dai et al. (2017b)^5^	Regression analysis using SAS V.9.3. *Confounders*: Age, sex, race, total energy intake; education attainment, annual household income, smoking status, physical activity and other dietary factors including dietary vitamin C (mg/day), K (μg/day), polyunsaturated fat (g/day), saturated fats, anti‐inflammatory drugs use, glycaemic load, DGAI‐2010 (Framingham study) and BMI.	*OAI* *Quartiles of total fibre (g/day): OR (95% CI), no. of cases* Q1 (8.6): 1.00 referent, 526/1970 Q2 (12.5): 0.98 (0.87, 1.10), 512/1988 Q3 (15.2): 0.96 (0.84, 1.08), 514/1994 Q4 (20.6): 0.85 (0.74, 0.99), 412/1999 *p*‐trend = 0.03 (significant)
			*OAI* *Quartiles of cereal fibre (g/day): OR (95% CI), no. of cases* Q1 (2.8): 1.00 referent, 554/1975 Q2 (4.5): 0.93 (0.83, 1.04), 474/1974 Q3 (6.0): 0.95 (0.84, 1.07), 480/1998 Q4 (8.4): 0.89 (0.79, 1.01), 456/2004 *p*‐trend = 0.07 (non‐significant)
			*OAI* *Quartiles of fruit and vegetable fibre (g/day): OR (95% CI) in OAI, no. of cases* Q1 (3.4): 1.00, referent, 496/1982 Q2 (5.8): 1.00 (0.89, 1.13), 508/1986 Q3 (7.9): 1.03 (0.90, 1.17), 512/1998 Q4 (11.5): 0.95 (0.82, 1.12), 448/1985 *p*‐trend = 0.77 (non‐significant)
			*OAI* *Quartiles of nut and legume fibre (g/day): OR (95% CI) in OAI, no. of cases* Q1 (0.5): 1.00, referent, 487/1980 Q2 (1.1): 1.04 (0.93, 1.17), 502/1982 Q3 (1.8): 1.04 (0.92, 1.17), 493/1979 Q4 (3.2): 1.00 (0.88, 1.13), 478/1998 *p*‐trend = 0.82 (non‐significant)
SxOA knee	Dai et al. (2017b)^5^	Regression analysis using SAS V.9.3. *Confounders*: Age, sex, race, total energy intake; education attainment, annual household income, smoking status, physical activity and other dietary factors including dietary vitamin C (mg/day), K (μg/day), polyunsaturated fat (g/day), saturated fats, anti‐inflammatory drugs use, glycaemic load, DGAI‐2010 (Framingham study) and BMI.	*Quartiles of total fibre (g/day): OR (95% CI) stratified by study, no. of cases* *OAI* Q1 (8.6): 1.00 referent, 208/1346 Q2 (12.5): 1.14 (0.89, 1.46), 256/1440 Q3 (15.1): 0.81 (0.62, 1.06), 206/1472 Q4 (20.6): 0.80 (0.60, 1.07), 199/1494 *p*‐trend = 0.03 (significant) *Framingham study* Q1 (13.7): 1.00, referent, 41/483 Q2 (14.8): 1.03 (0.53, 2.00), 50/488 Q3 (19.1): 0.46 (0.22, 0.95), 24/484^a^ Q4 (25.5): 0.39 (0.17, 0.92), 28/486^a^ *p*‐trend = 0.03 (significant)
			*Quartiles of cereal fibre (g/day): OR (95% CI) stratified by study, no. of cases* *OAI* Q1 (2.8): 1.00 referent, 211/1348 Q2 (4.5): 1.05 (0.83, 1.34), 226/1420 Q3 (6.0): 1.00 (0.79, 1.28), 215/1450 Q4 (8.4): 0.96 (0.75, 1.24), 217/1534 *p*‐trend = 0.79 (non‐significant) *Framingham study* Q1 (3.7): 1.00, referent, 38/453 Q2 (4.4): 1.28 (0.70, 2.33), 46/457 Q3 (5.8): 0.62 (0.32, 1.20), 25/454 Q4 (9.7): 0.57 (0.28, 1.17), 26/454 *p*‐trend = 0.06 (non‐significant)
			*Quartiles of fruit and vegetable fibre (g/day): OR (95% CI) stratified by study, no. of cases* *OAI* Q1 (3.4): 1.00 referent, 214/1374 Q2 (5.8): 1.00 (0.78, 1.28), 238/1504 Q3 (7.9): 0.89 (0.68, 1.17), 205/1434 Q4 (11.5): 0.83 (0.60, 1.15), 212/1440 *p*‐trend = 0.24 (non‐significant) *Framingham study* Q1 (3.6): 1.00 referent, 39/453 Q2 (5.8): 0.17 (0.07, 0.44), 16/456^a^ Q3 (8.3): 0.80 (0.35, 1.79), 52/453 Q4 (12.8): 0.44 (0.16, 1.23), 28/456 *p*‐trend = 0.57 (non‐significant)
			*Quartiles of nut and legume fibre (g/day): OR (95% CI) stratified by study, no. of cases* *OAI* Q1 (0.5): 1.00, referent, 196/1378 Q2 (1.1): 1.08 (0.84, 1.39), 222/1436 Q3 (1.8): 1.08 (0.84, 1.39), 230/1446 Q4 (3.2): 1.03 (0.79, 1.34), 220/1484 *p*‐trend = 0.99 (non‐significant) *Framingham study* Q1 (0.7): 1.00, referent, 40/453 Q2 (1.6): 0.59 (0.31, 1.14), 37/457 Q3 (2.4): 0.29 (0.14, 0.59), 24/452^a^ Q4 (4.4): 0.41 (0.20, 0.82), 34/456^a^ *p*‐trend = 0.03 (significant)

^a^ Data with strong effects.

Abbreviations: aMED, Mediterranean Diet Score; BMI, Body Mass Index; CES‐D, Centre for Epidemiologic Studies Depression Scale; CI, confidence interval; DGAI‐2010, Dietary Guidelines Adherence Index; E‐DII, Energy‐Adjusted Dietary Inflammatory Index; g/day, grams per day; JSW, joint space width; KL, Kellgren–Lawrence; KOA, knee osteoarthritis; no., number; OA, osteoarthritis; OAI, osteoarthritis initiative; OR, odds ratio; PASE, physical activity scale for the elderly; QoL, quality of life; RR, relative risk; SAS, statistical analysis software; SxOA, symptomatic osteoarthritis; VAS, visual analog scale; WOMAC, Western Ontario and McMaster Universities Osteoarthritis Index.

**TABLE 4:** Joanna Briggs Institute critical appraisal results

	Dai et al. (2017a)^37^	Dai et al. (2017b)^5^	Veronese et al. (2019)^38^	Liu et al. (2020)^34^	Xu et al. (2020)^4^	Ruan et al. (2021)^39^
Were the two groups similar and recruited from the same population?	N	N	N	N	N	N
Were the exposures measured similarly to assign people to both exposed and unexposed groups?	Y	Y	Y	Y	Y	Y
Was the exposure measured in a valid and reliable way?	Y	Y	Y	Y	Y	Y
Were confounding factors identified?	Y	Y	Y	Y	Y	Y
Were strategies to deal with confounding factors stated?	Y	Y	Y	Y	Y	Y
Were the groups/participants free of the outcome at the start of the study (or at the moment of exposure)?	NA	NA	NA	NA	NA	NA
Were the outcomes measured in a valid and reliable way?	Y	Y	Y	Y	Y	Y
Was the follow up time reported and sufficient to be long enough for outcomes to occur?	Y	Y	Y	Y	Y	Y
Was follow up complete, and if not, were the reasons to loss to follow up described and explored?	Y	Y	Y	Y	Y	Y
Were strategies to address incomplete follow up utilised?	Y	N	N	N	Y	Unclear
Was appropriate statistical analysis used?	Y	Y	Y	Y	Y	Y
Overall	Include	Include	Include	Include	Include	Include

The results of the review were heterogeneous, and no studies explored the association of the same dietary pattern with the same outcome. The outcomes grouped by dietary pattern and certainty of evidence are presented in Table 3.

Two studies evaluated overall diet quality effects on symptomatic osteoarthritis.^4^, ^39^ WOMAC and Visual Analog Scale scores were evaluated using Australian Recommended Food Score associations in the Vitamin D Effect on Osteoarthritis (VIDEO) study conducted on male and female participants between 2010 and 2013 with a 24‐month follow up. After adjusting for confounders, Australian Recommended Food Score was not significantly associated with WOMAC knee pain, joint stiffness, physical dysfunction and knee pain Visual Analog Scale over 24 months.^39^

The effects of diet quality on symptomatic osteoarthritis were evaluated via the American Osteoarthritis Initiative from February 2004 to May 2006 in male and female participants aged 45–79 years, using a validated 70‐item Block Brief Food Frequency Questionnaire measuring diet quality.^4^ A significant negative effect of increasing poor diet quality was observed on symptomatic knee osteoarthritis progression at 24‐months follow up after adjusting for confounders (*p*‐trend <0.01).

The effects of dietary inflammatory potential on symptomatic knee osteoarthritis were evaluated using a validated Block brief 2000 food frequency questionnaire using the American Osteoarthritis Initiative cohort, with a 48‐month follow up.^34^ Participants were stratified by quartiles of Energy‐density Dietary Inflammatory Index score; OR (95%). A linear statistically‐significant relationship was observed between a higher pro‐inflammatory diet and symptomatic knee osteoarthritis (OR 1.43 [1.16, 1.76] *p* = 0.001).

The effects of Western dietary patterns and Prudent dietary patterns on symptomatic osteoarthritis progression were evaluated using American Osteoarthritis Initiative data.^4^ Symptomatic osteoarthritis progression significantly increased with higher compliance of Western patterns represented by higher quartiles, with greater intakes of red and/or processed meats, refined grains and potato chips (*p*‐trend <0.01). Symptomatic osteoarthritis progression significantly reduced with increased adherence to Prudent dietary patterns of high vegetable, fruit, fish, wholegrain and legume intake, represented by higher quartiles of intake compared to lower intakes (*p*‐trend <0.01).

The effects of the Mediterranean Diet on symptomatic osteoarthritis and pain worsening was evaluated using the American Osteoarthritis Initiative cohort using the Block Brief 2000 food frequency questionnaire to calculate Mediterranean Diet scores over a 48‐month follow up.^38^ Participants in quintile 5 with greater adherence to the Mediterranean Diet reported a significantly lower pain worsening and symptomatic osteoarthritis progression risk, and an increase in one standard deviation of the Mediterranean Diet score reduced the pain worsening and symptomatic osteoarthritis risk. However, the trend was inconclusive.

The effects of dietary fibre on WOMAC pain were evaluated using the Block Brief 2000 food frequency questionnaire using the American Osteoarthritis Initiative cohort with a 96‐month follow up.^37^ Statistically‐significant effects were observed between higher total fibre and fruit and vegetable fibre intake and moderate knee pain and between higher total, cereal and fruit and vegetable fibre intake and severe knee pain.

Associations between dietary fibre and incident symptomatic osteoarthritis and pain worsening were evaluated using the American Osteoarthritis Initiative cohort between 2004 and 2006 and Framingham Offspring Cohort with a 9.5 year follow up.^5^ In the American Osteoarthritis Initiative, pain worsening was inversely associated with total fibre intake (*p* = 0.03). However, no significant effects were observed between each fibre subtype and pain worsening in the American Osteoarthritis Initiative. In the Framingham Study, total fibre intake was inversely associated with symptomatic osteoarthritis incidence. Moderate fruit and vegetable fibre intake and high nut and legume fibre intake had reduced symptomatic osteoarthritis incidence.

The certainty of the evidence was very low for symptomatic osteoarthritis progression, knee pain visual analog scale, WOMAC joint stiffness, WOMAC knee pain, total WOMAC, WOMAC physical dysfunction and WOMAC pain. The certainty of the evidence was low for pain worsening (Table 5).

**TABLE 5:** Assessment of the certainty of the evidence using the GRADE system

Certainty assessment							Certainty
No. of studies	Study design	Risk of bias	Inconsistency	Indirectness	Imprecision	Other considerations	
*Symptomatic osteoarthritis progression*							
4^4^, ^5^, ^34^, ^38^	Observational studies	Serious^a^	Not serious	Not serious	Serious^b^	Dose response gradient	⨁◯◯◯ Very low
*Knee pain VAS*							
1^39^	Observational studies	Serious^a^	Not serious	Not serious	Serious^c^	None	⨁◯◯◯ Very low
*WOMAC joint stiffness*							
1^39^	Observational studies	Serious^a^	Not serious	Not serious	Serious^c^	None	⨁◯◯◯ Very low
*WOMAC knee pain*							
1^39^	Observational studies	Serious^a^	Not serious	Not serious	Serious^c^	None	⨁◯◯◯ Very low
*Total WOMAC*							
1^39^	Observational studies	Serious^a^	Not serious	Not serious	Very serious^d^	None	⨁◯◯◯ Very low
*WOMAC physical dysfunction*							
1^39^	Observational studies	Serious^a^	Not serious	Not serious	Very serious^d^	None	⨁◯◯◯ Very low
*Pain worsening*							
2^5^, ^38^	Observational studies	Serious^a^	Not serious	Not serious	Not serious	Dose response gradient	⨁⨁◯◯ Low
*WOMAC pain*							
1^37^	Observational studies	Serious^a^	Not serious	Not serious	Serious^e^	None	⨁◯◯◯ Very low

Abbreviation: CI, confidence interval; VAS, Visual Analog Scale; WOMAC, Western Ontario and McMaster Universities Osteoarthritis Index.

^a^ Cohort study design without concealment to data analysis, significantly different baseline participant characteristics across exposure groups or no statistical analysis to evaluate baseline participant characteristic differences, and unclear strategies utilised to address incomplete follow up.

^b^ The associations between dietary inflammatory potential and symptomatic osteoarthritis and Western diet intake and symptomatic osteoarthritis, were downgraded for serious imprecision due to wide confidence intervals which include appreciable harm. The association between poor diet quality and symptomatic osteoarthritis was not downgraded for imprecision as the confidence interval did not cross appreciable harms and null effects. The association between Prudent diet and symptomatic osteoarthritis progression, between the Mediterranean Diet and symptomatic osteoarthritis, and between and dietary fibre and symptomatic osteoarthritis had no imprecision downgrades, as confidence intervals for the highest adherence of those diet did not cross appreciable benefit or harm.

^c^ Serious imprecision due to wide confidence intervals spanning appreciable benefit and harm.

^d^ Very serious imprecision due to very wide confidence intervals crossed appreciable benefits, null effects and appreciable harms.

^e^ The association between each fibre category and mild, moderate and severe knee pain was downgraded for very serious imprecision due to wide confidence intervals.

The overall risk of bias was very serious across the six studies. The VIDEO^39^ and American Osteoarthritis Initiative studies^4^, ^34^, ^37^, ^38^ were downgraded due to cohort study design without concealment to data analysis, significantly different baseline participant characteristics across exposure groups or no statistical analysis to evaluate baseline participant characteristic differences, and unclear strategies utilised to address incomplete follow up (Table 3). The study that compared the results of American Osteoarthritis Initiative and Framingham Offspring Cohorts^5^ was downgraded due to cohort study design, lack of baseline participant characteristic statistical comparison and unclear strategies to address incomplete follow‐up (Table 3).

The imprecision of individual studies varied (Table 3). The associations between diet quality and WOMAC knee pain, joint stiffness and knee pain Visual Analog Scale had wide CIs spanning appreciable benefit and harm, thus were downgraded for serious imprecision.^39^ The association between diet quality and total WOMAC and WOMAC physical dysfunction were downgraded due to very serious imprecision, as very wide CIs crossed appreciable benefits, null effects and appreciable harms.^39^ The association between poor diet quality and symptomatic osteoarthritis was not downgraded for imprecision as the CI did not cross appreciable harms and null effects.^4^ The association between dietary inflammatory potential and symptomatic osteoarthritis was downgraded for serious imprecision due to wide CIs for higher quartiles.^34^

Furthermore, the association between Western dietary patterns and symptomatic osteoarthritis progression was downgraded for imprecision due to wide CIs.^4^ The association between Prudent diet and symptomatic osteoarthritis progression was not downgraded for imprecision.^4^ The association between the Mediterranean Diet and symptomatic osteoarthritis and pain worsening had no imprecision downgrades, as CIs for all scores crossed the null effect, but did not reach appreciable benefit or harm.^38^ The association between each fibre category and mild, moderate and severe knee pain was downgraded for very serious imprecision due to wide CIs.^37^ The associations between dietary fibre and incident symptomatic osteoarthritis and pain worsening were not downgraded for imprecision^5^.

The eligible studies examined the effect of different dietary patterns or food groups on the progression of symptomatic osteoarthritis or the change of osteoarthritis‐specific symptoms within a period of time. Due to the measurement differences of dietary patterns, food groups and outcomes, it was not possible to conduct meta‐analysis to pool the data for the overall effect. Therefore, the overall relative risk and CI were not calculated due to the variance of exposure and outcome. Thus, the overall imprecision could not be assessed.

In the included articles, the exposure included either dietary patterns or food groups using different types of food frequency questionnaires, such as Block Brief 2000 food frequency questionnaire,^5^, ^34^, ^37^, ^38^ Dietary Questionnaire for Epidemiological Studies v2,^39^ 70‐item Block Brief food frequency questionnaire.^4^ Moreover, the definitions of the outcome varied across all studies, including prevalence of different level of pain,^37^ pain and/or function worsening,^4^, ^5^, ^38^, ^39^ and new onset of symptomatic osteoarthritis.^5^, ^34^, ^38^ As a result, the exposure and the outcome of each article were not able to be compared directly, and the statistical assessment for analysing heterogeneity was not applicable. Therefore, the different effects across studies and the overall inconsistency could not be assessed.

Based on the eligibility criteria, all included articles were strictly aligned with the study question regarding population, intervention/exposure, comparison and outcomes (PICO). Hence, the evidence in included papers can apply and address the research question of this systematic review, suggesting little indirectness.

The results of the included articles presented positive, null and negative effects for different dietary patterns, so the publication bias was considered as undetected. However, publication bias cannot be ruled out as only articles in English were eligible for this systematic review. There may be non‐English studies that complied with the inclusion criteria, however, were excluded due to the limitation of reading other languages.

## 4 DISCUSSION

This systematic review consolidates available evidence on the associations between dietary habits, diet quality, food groups and symptomatic osteoarthritis in adults aged 45 years and older with joint pain, aching or stiffness. Poor diet quality was associated with accelerated symptomatic osteoarthritis progression,^4^ whereas healthy diets aligned with the Dietary Guidelines for Australian Adults and Australian Guide to Healthy Eating had an inconclusive effect.^39^

In terms of dietary pattern, diets with a higher inflammatory potential were linked to a higher incidence of symptomatic osteoarthritis.^34^ The Western pattern had an increased effect on symptomatic osteoarthritis advancement, whereas the Prudent pattern had a reduced effect.^4^ The Mediterranean dietary pattern reduced knee symptomatic osteoarthritis progression and pain worsening.^38^ In addition, total dietary fibre and fibre from different food groups had null, reduction and inconclusive effects on pain worsening in different pain categories.^5^, ^37^

Only six articles from three cohort studies were included. The certainty of the body of evidence ranged from low to very low and is limited. As highlighted, little is known about diet quality in individuals with osteoarthritis and its interrelationship with pain, physical dysfunction and quality of life,^42^ indicating a need for additional research of a higher certainty of evidence.

Two studies explored associations between overall diet quality and symptomatic osteoarthritis, however, were inconsistent. One study found no significant association between diet quality and symptomatic osteoarthritis,^39^ while the other study found a positive association between poor diet quality and symptomatic osteoarthritis.^4^ The inconclusive results could be attributed to a few factors, one being the different sample size (392 participants in VIDEO^39^ against 2757 participants in American Osteoarthritis Initiative^4^). The other factor could be the variation in how diet quality score is calculated. One study included alcohol when calculating the diet quality score,^39^ whereas the other adjusted alcohol intake as a confounder.^4^ A systematic review suggested alcohol may contribute to osteoarthritis via the mediation of BMI, despite its inconclusive effects.^43^

The Western dietary pattern was positively associated with progression of symptomatic osteoarthritis.^4^ The Western diet is abundant in saturated fat intake, which promotes white adipose tissue expansion and adipocyte dysfunction.^44^, ^45^ In addition, high energy and high refined carbohydrate intake are also the main components of the Western Diet.^44^ These components contribute to the enhancement of inflammatory signalling and activation of inflammatory gene expression, hence increasing inflammation.^44^, ^45^ A prior study established a strong positive correlation between Western dietary patterns and the dietary inflammatory index.^46^ Greater dietary inflammatory Index scores are linked with increased weight gain, obesity risk,^47^ greater pain severity^42^ and knee osteoarthritis prevalence.^34^ However, associations between DII and symptomatic osteoarthritis may not entirely be accounted for via effect on BMI, and is potentially mediated via inflammatory markers.^34^, ^48^ Therefore, the Western diet and the pro‐inflammatory diet may have similar mechanisms in accelerating osteoarthritis progression. However, additional research is needed to determine the degree of impacts of the Western diet and diets with high inflammatory potential using consistent symptomatic osteoarthritis outcome assessments. These include using WOMAC pain or pain improvement scores, or the same definition of symptomatic osteoarthritis, such as using the WOMAC score.

One study explored Mediterranean diet associations on symptomatic osteoarthritis and pain worsening,^38^ overall drawing inconclusive effects. Increased adherence to a Mediterranean diet may alleviate osteoarthritis symptoms by lowering serum levels of pro‐inflammatory cytokines and other mediators, such as high‐sensitivity C‐reactive protein, interleukins 6, 7 and 18, as well as decreasing oxidative stress biomarkers.^49^, ^50^, ^51^ Thus, reducing these biomarkers slows cartilage degeneration.^7^, ^52^, ^53^ Greater Mediterranean Diet scores were associated with reduced inflammation,^50^, ^54^ oxidative stress^47^ and greater fibre and vitamins, which may exhibit a protective effect on osteoarthritis outcomes.^5^ The protective effects of the Mediterranean diet on the prevalence of osteoarthritis were shown in a previous systematic review with a fair risk of bias in middle‐aged adults and the elderly with or at risk of osteoarthritis.^33^

The Prudent diet drew decreased effects on symptomatic osteoarthritis. The protective benefits of the Prudent dietary pattern on the risk of knee osteoarthritis were explored in adults aged over 45 years at a good quality in a previous systematic review.^32^ This study^32^ was a meta‐analysis exploring associations between general symptomatic osteoarthritis, knee osteoarthritis and dietary patterns. It included only one article, which had symptomatic osteoarthritis as a primary outcome. Given the main aim of this research investigating dietary patterns and symptomatic osteoarthritis, the current study's results will add further value to the previous meta‐analysis.^32^

Dietary components of the Prudent diet are similar to that of the Mediterranean diet, generally including high consumption of vegetables, fruits, fish, whole grains and legumes.^39^ In addition, high adherence to a prudent diet was negatively associated with systemic inflammatory biomarkers, such as leptin, soluble intracellular adhesion molecule 1, E‐selectin and C‐reactive protein.^55^, ^56^ As such, the causes for the preventive effects of decreasing symptomatic osteoarthritis progression of the Prudent diet may be similar to those of the Mediterranean diet via similar mechanisms explained above.

Two studies examined the associations between dietary fibre intake and symptomatic osteoarthritis.^5^, ^37^ The protective effects of total fibre consumption were constant across both studies with a significant dose‐dependent downward trend, however, the effects of fibre from each food group were inconclusive. The protective effects of dietary fibre are regulated by its fermentation products, short chain fatty acids.^57^ Short chain fatty acids inhibit inflammatory responses by activating G Protein‐coupled Receptor,^58^ which has anti‐inflammatory properties, and by suppressing the expression of pro‐inflammatory cytokines.^57^, ^59^ One study discovered that fruit and vegetable fibre were inversely associated with moderate knee pain and cereal fibre and fruit and vegetable fibre were inversely associated with severe knee pain,^37^ whereas the other failed to demonstrate significant effects of fibre from each food group.^5^ One study included an additional adjustment for BMI,^5^ whilst the other did not.^37^ Another argument could be that categorising pain levels allows for more precise outcomes, as the magnitude of positive effects may be diluted in the absence of pain classification. One study^37^ explored fibre intake effects on mild, moderate and severe pain, whilst one investigated effects on overall pain.^5^ Moreover, increased total fibre, and nut and legume fibre, were found to be inversely associated with incidence symptomatic osteoarthritis in the Framingham study, such effects attenuated in American Osteoarthritis Initiative.^5^ The discrepancy could be explained by the fact that American Osteoarthritis Initiative and the Framingham trial had different sample sizes, participant characteristics, especially that the American Osteoarthritis Initiative cohort had a higher mean BMI than Framingham Study participants, raising collider bias potential via conditioning on BMI, and dietary fibre quartile cut‐offs and the Framingham study measured dietary consumption 4 years later at follow‐up. Slight intake increments present in the study sample over the follow‐up were unaccounted for in the American Osteoarthritis Initiative cohort.^37^ Furthermore, additional research focusing on dietary fibre intake and associations with symptomatic osteoarthritis in adults 45+ year would be beneficial to raise the certainty of the body of evidence.

In contrast to previous review studies, the present systematic review focused exclusively on symptomatic osteoarthritis as the outcome. The findings indicate that diet quality, dietary patterns and food groups influence not just radiographic osteoarthritis progression, but also symptomatic osteoarthritis progression. Many previous studies explored nutrient and symptomatic osteoarthritis associations, with single dietary components failing to be corroborated in randomised trials.^20^ Review strengths included the large sample size of the six articles, PROSPERO registration to allow method transparency, a comprehensive search strategy that was reviewed by supervisors and University of Sydney librarians, and included five databases. Authors were contacted to access full‐text of articles. Quality assessment tools enabled risk of bias assessment for cohort studies using Joanna Briggs Institute checklists. Certainty of the evidence for dietary patterns and outcomes were assessed using GRADE. 

However, there were limitations. Bi‐direction via meta‐analysis could not be explored due to the few included studies and heterogeneity of exposure and outcome measurements. A range of dietary patterns and food groups were included using different measuring methods. Despite the fact that all dietary intake measurement methods are validated, their accuracy may be varied. Knee pain assessment heterogeneity between studies may also hinder the review's accuracy of findings. Frequent knee pain was defined as pain, aching or stiffness in the knee on most days during the past 30 days,^5^, ^38^ whilst one study queried whether participants had experienced this over the past 12 months.^34^ Large effects were identified as more than 50% of protective effects observed for dietary patterns on symptomatic osteoarthritis outcomes. Upgrading the certainty of evidence in this review was prohibited due to concerns about risk of bias and imprecision.^60^, ^61^ The risk of bias of the included studies cannot be eliminated due to potential residual confounders in the observational studies, such as BMI,^34^, ^37^ alcohol^5^, ^34^, ^37^, ^39^ and depression,^5^, ^34^, ^39^ cohort study design, significant differences among baseline participant characteristics baseline and unclear strategies addressing loss to follow up. Additionally, studies merely assessed baseline dietary intake, with the exception of the Framingham study.^5^ Under‐ or overestimation of dietary intake can occur due to self‐reported dietary data prone to bias.^62^ Cohort studies are observational studies that do not prove causality, raising residual confounding concerns.^5^ They merely provide empirical evidence, and results should be confirmed by studies at higher certainty of evidence.

There are limitations regarding search strategy, language and practicality. This systematic review included only English‐language articles and lacked exhaustive search strategies for trial registries and grey literature from websites and organisations. Additionally, despite attempts to contact authors for full‐text retrieval, certain articles were excluded due to author non‐response. Therefore, it is plausible that certain pertinent articles may be omitted. Furthermore, studies involving intervention via energy restriction were not included. This is because the effect of diet is difficult to distinguish from the effect of weight loss. Finally, because all six studies involved participants with osteoarthritis and were conducted in developed countries, the findings of this systematic review may not be generalisable to non‐American Osteoarthritis Initiative populations as dietary patterns vary by ethnic and environmental backgrounds.

As shown by the results of the six reviewed studies, associations were identified between dietary intake, diet quality, food groups and symptomatic osteoarthritis in adults aged 45 years and older. Participants following a higher prudent dietary pattern had the greatest symptomatic osteoarthritis reduction. The currently limited body of evidence due to low certainty attributable to heterogeneity and study limitations suggest that there is a knowledge gap regarding the association between diet and symptomatic osteoarthritis. Further research is warranted to confirm the estimated effects at a high certainty of evidence, and to investigate the effects of other dietary patterns and food groups on symptomatic osteoarthritis, such as DASH. Identifying the most effective dietary patterns may aid in the development of future symptomatic osteoarthritis management guidelines.

## AUTHOR CONTRIBUTIONS

AD and VH conceptualised the systematic review. DF and JZ contributed to literature search screening, data extraction, quality assessment and draft manuscript. VH and AD contributed to initial research question development, methodology, quality assessment and data extraction, as well as providing critical feedback on the manuscript. The final manuscript was reviewed and approved by all authors. The authors acknowledge the University of Sydney Librarians for their assistance with reviewing databases and the search strategy.

## CONFLICT OF INTEREST

Vasant Hirani is Associate Editor of Nutrition & Dietetics. They were excluded from the peer review process and all decision‐making regarding this article. This manuscript has been managed throughout the review process by the Journal's Editor‐in‐Chief. The Journal operates a blinded peer review process and the peer reviewers for this manuscript were unaware of the authors of the manuscript. This process prevents authors who also hold an editorial role to influence the editorial decisions made. Other authors have no conflicts of interest to declare.

## Supporting information


**Data S1**: Supporting Information

## Data Availability

Data sharing is not applicable to this article as no new data were created or analysed in this study.
