# Supplementary material for: The effects of dietary patterns and food groups on symptomatic osteoarthritis: A systematic review
Source: Nutr Diet. 2022 Oct 24;80(1):21–43. doi: 10.1111/1747-0080.12781 (PMC10092134; doi:10.1111/1747-0080.12781)
Supplement: Supplementary file 1 — Data S1: Supporting Information [file NDI-80-21-s001.pdf]

PRISMA checklist

Section and Topic	Item #	Checklist item	Location where item is reported
**TITLE**			
**Title**	**1**	**Identify the report as a systematic review.**	1
**ABSTRACT**			
**Abstract**	**2**	**See the PRISMA 2020 for Abstracts checklist.**	**1,2**
**INTRODUCTION**			
**Rationale**	**3**	**Describe the rationale for the review in the context of existing knowledge.**	**2 - 4**
**Objectives**	**4**	**Provide an explicit statement of the objective(s) or question(s) the review addresses.**	**4**
**METHODS**			
**Eligibility criteria**	**5**	**Specify the inclusion and exclusion criteria for the review and how studies were grouped for the syntheses.**	**6**
**Information sources**	**6**	**Specify all databases, registers, websites, organisations, reference lists and other sources searched or consulted to identify studies. Specify the date when each source was last searched or consulted.**	**5**

**Search strategy**	**7**	**Present the full search strategies for all databases, registers and websites, including any filters and limits used.**	**6 and Table S2**
**Selection process**	**8**	**Specify the methods used to decide whether a study met the inclusion criteria of the review, including how many reviewers screened each record and each report retrieved, whether they worked independently, and if applicable, details of automation tools used in the process.**	**6 and Table 1**
**Data collection process**	**9**	**Specify the methods used to collect data from reports, including how many reviewers collected data from each report, whether they worked independently, any processes for obtaining or confirming data from study investigators, and if applicable, details of automation tools used in the process.**	**6**
**Data items**	**10a**	**List and define all outcomes for which data were sought. Specify whether all results that were compatible with each outcome domain in each study were sought (e.g. for all measures, time points, analyses), and if not, the methods used to decide which results to collect.**	**Table 2**
	**10b**	**List and define all other variables for which data were sought (e.g. participant and intervention characteristics, funding sources). Describe any assumptions made about any missing or unclear information.**	**6 and Table 2**
**Study risk of bias assessment**	**11**	**Specify the methods used to assess risk of bias in the included studies, including details of the tool(s) used, how many reviewers assessed each study and whether they worked independently, and if applicable, details of automation tools used in the process.**	**6 -7**
**Effect measures**	**12**	**Specify for each outcome the effect measure(s) (e.g. risk ratio, mean difference) used in the synthesis or presentation of results.**	**Table 3**
**Synthesis methods**	**13a**	**Describe the processes used to decide which studies were eligible for each synthesis (e.g. tabulating the study intervention characteristics and comparing against the planned groups for each synthesis (item #5)).**	**Table 2**
	**13b**	**Describe any methods required to prepare the data for presentation or synthesis, such as handling of missing summary statistics, or data conversions.**	**NA**

	13c	**Describe any methods used to tabulate or visually display results of individual studies and syntheses.**	6
	13d	**Describe any methods used to synthesize results and provide a rationale for the choice(s). If meta-analysis was performed, describe the model(s), method(s) to identify the presence and extent of statistical heterogeneity, and software package(s) used.**	N/A
	13e	**Describe any methods used to explore possible causes of heterogeneity among study results (e.g. subgroup analysis, meta-regression).**	N/A
	13f	**Describe any sensitivity analyses conducted to assess robustness of the synthesized results.**	N/A
**Reporting bias assessment**	14	**Describe any methods used to assess risk of bias due to missing results in a synthesis (arising from reporting biases).**	6 - 7
**Certainty assessment**	15	**Describe any methods used to assess certainty (or confidence) in the body of evidence for an outcome.**	7
**RESULTS**			
**Study selection**	16a	**Describe the results of the search and selection process, from the number of records identified in the search to the number of studies included in the review, ideally using a flow diagram.**	7, 8
	16b	**Cite studies that might appear to meet the inclusion criteria, but which were excluded, and explain why they were excluded.**	8
**Study characteristics**	17	**Cite each included study and present its characteristics.**	8 - 9

**Risk of bias in studies**	**18**	**Present assessments of risk of bias for each included study.**	**9**
**Results of individual studies**	**19**	**For all outcomes, present, for each study: (a) summary statistics for each group (where appropriate) and (b) an effect estimate and its precision (e.g. confidence/credible interval), ideally using structured tables or plots.**	**10-15**
**Results of syntheses**	**20a**	**For each synthesis, briefly summarise the characteristics and risk of bias among contributing studies.**	**11 -15**
	**20b**	**Present results of all statistical syntheses conducted. If meta-analysis was done, present for each the summary estimate and its precision (e.g. confidence/credible interval) and measures of statistical heterogeneity. If comparing groups, describe the direction of the effect.**	**N/A**
	**20c**	**Present results of all investigations of possible causes of heterogeneity among study results.**	**10**
	**20d**	**Present results of all sensitivity analyses conducted to assess the robustness of the synthesized results.**	**N/A**
**Reporting biases**	**21**	**Present assessments of risk of bias due to missing results (arising from reporting biases) for each synthesis assessed.**	**10-11**
**Certainty of evidence**	**22**	**Present assessments of certainty (or confidence) in the body of evidence for each outcome assessed.**	**11**
**DISCUSSION**			
**Discussion**	**23a**	**Provide a general interpretation of the results in the context of other evidence.**	**16 - 22**
	**23b**	**Discuss any limitations of the evidence included in the review.**	**21**

	**23c**	**Discuss any limitations of the review processes used.**	**21**
	**23d**	**Discuss implications of the results for practice, policy, and future research.**	**22**
**OTHER INFORMATION**			
**Registration and protocol**	**24a**	**Provide registration information for the review, including register name and registration number, or state that the review was not registered.**	**Title page**
	**24b**	**Indicate where the review protocol can be accessed, or state that a protocol was not prepared.**	**N/A**
	**24c**	**Describe and explain any amendments to information provided at registration or in the protocol.**	**N/A**
**Support**	**25**	**Describe sources of financial or non-financial support for the review, and the role of the funders or sponsors in the review.**	**N/A**
**Competing interests**	**26**	**Declare any competing interests of review authors.**	**Title page**
**Availability of data, code and other materials**	**27**	**Report which of the following are publicly available and where they can be found: template data collection forms; data extracted from included studies; data used for all analyses; analytic code; any other materials used in the review.**	**N/A**

Table S2. Search Strategy

Ovid MEDLINE(R) ALL <1946 to August 30, 2021>

1 Middle Aged/ 4566121

2 exp Aged/ 3293845

3 exp Aging/ 255541

4 middle age*.tw.54653

5 ((Adult* or population* or people* or individual* or person*) adj2 (old* or age* or elder*)).tw. 338517

6 ((Adult* or population* or people* or individual* or person*) adj2 middle age*).tw. 9400

7 ((More or over or greater) adj2 (45 year* or 45 yr*)).tw. 2423

8 age*.tw. 4007948

9 elder*.tw. 273956

10 old*.tw. 1579477

11 1 or 2 or 3 or 4 or 5 or 6 or 7 or 8 or 9 or 10 8678446

12 (symptom* adj2 disease*).tw. 23553

13 symptom*.tw. 1245577

14 (symptom* adj2 osteoarthrit*).tw. 1743

15 exp Osteoarthritis/ 68338

16 exp Cartilage/ 88963

17 exp Meniscus/ 7798

18 Bone Marrow/ 68961

19 Osteophyte/ 923

20 Synovitis/ 7639

21 exp Ligaments/ 42044

22 osteoarthrit*.tw. 73353

23 cartilag*.mp. 119271

24 chondral.mp. 4086

25 meniscal.mp. 8591

26 meniscus.mp. 13704

27 bone marrow*.mp. 278890

28 subchondral.mp. 8463

29 osteophyte*.mp. 5099

30 effus*.mp. 68705

31 synovitis.mp. 14990

32 ligament*.mp. 97295

33 fat pad*.mp. 7772

34 attrit*.mp. 15072

35 12 or 13 or 14 or 15 or 16 or 17 or 18 or 19 or 20 or 21 or 22 or 23 or 24 or 25 or 26 or 27 or 28 or 29 or 30 or 31 or 32 or 33 or 34 1875462

36 exp Diet/ 300287

37 exp Food/ 1353515

38 (diet* adj3 (score* or pattern* or intake* or treatment* or quality)).tw. 89158

39 (meal* adj3 (score* or pattern* or intake* or treatment* or quality)).tw. 4597

40 (food* adj3 (score* or pattern* or intake* or treatment* or quality)).tw. 72378

41 (pattern* adj3 (diet* or eat* or meal* or food*)).tw. 19097

42 ((eat* or diet* or meal*) adj3 habit*).tw. 21107

43 diet*.tw. 602641

44 food*.mp. 683449

45 food group*.mp. 5860

46 (diet* adj3 (artificial or atkins or carb* or carb* loading or cariogenic or casein free or cereal base* or DASH or element* or eliminat* or experiment* or fib* free or gluten free or healthy or high calorie* or high fib* or high glycemic index or high GI or high salt or high sodium or intuitive eat* or keto* or lactose free or lipid* or liquid or low calorie* or low carb* or low fib* or low FODMAP or low glycemic index or low GI or low iodine or low residue* or macrobiotic or Mediterranean or nordic or obesogen* or paleo* or protein or raw food or soft or unhealthy or vegetarian or vegan or western*)).mp. 132580

47 36 or 37 or 38 or 39 or 40 or 41 or 42 or 43 or 44 or 45 or 46 2194230

48 Pain/ 138564

49 Musculoskeletal Pain/ 3773

50 Chronic Pain/ 17549

51 pain*.mp. 848357

52 ach*.mp. 1246773

53	stiff*.mp.	90092
54	tender*.mp.	25765
55	tight*.mp.	136756
56	swell*.mp.	103272
57	(symptom* adj3 (pain or stiff* or tender* or tight* or swell*)).mp.	28573
58	exp Joints/	258410
59	joint*.tw.	343335
60	48 or 49 or 50 or 51 or 52 or 53 or 54 or 55 or 56 or 57	2334166
61	58 or 59	495254
62	11 and 35 and 47 and 60 and 61	721

Embase Classic+Embase <1947 to 2021 August 30>

1 middle aged/ 1928837

2 aged/ 3382184

3 frail elderly/ 10955

4 very elderly/ 239083

5 middle age*.tw.73992

6 ((More or over or greater) adj2 (45 year* or 45 yr*)).tw. 3866

7 ((Adult* or population* or people* or individual* or person*) adj2 (old* or age* or elder*)).tw. 461870

8 ((Adult* or population* or people* or individual* or person*) adj2 middle age*).tw. 12703

9 age*.tw. 6113823

10 elder*.tw. 399421

11 old*.tw. 2349478

12 1 or 2 or 3 or 4 or 5 or 6 or 7 or 8 or 9 or 10 or 11 10184253

13 symptom/ 151812

14 (symptom* adj2 disease*).tw. 34593

15 symptom*.tw. 1948700

16 (symptom* adj2 osteoarthrit*).tw. 2625

17 exp osteoarthritis/ 149696

18 knee meniscus/ 11590

19 bone marrow/ 159295

20 subchondral bone/ 1478

21 subchondral bone plate/ 448

22 osteophyte/ 9119

23 exp synovitis/ 28625

24 fat pad/2272

25 effusion/ 17162

26 joint effusion/ 6025

27 ligament/ 33762

28 exp joint ligament/ 29077

29 cartilage/ 55565

30 osteoarthriti*.tw. 108565

31 cartilag*.mp. 173553

32 chondral.mp. 5382

33 meniscal.mp. 11960

34 meniscus.mp. 23131

35 bone marrow*.mp. 516860

36 subchondral.mp. 12342

37 osteophyte*.mp. 11380

38 effus*.mp. 144607

39 synovitis.mp. 27868

40 ligament*.mp. 133593

41 fat pad*.mp. 13022

42 attrit*.mp. 19107

43 13 or 14 or 15 or 16 or 17 or 18 or 19 or 20 or 21 or 22 or 23 or 24 or 25 or 26 or 27 or 28 or 29 or 30 or 31 or 32 or 33 or 34 or 35 or 36 or 37 or 38 or 39 or 40 or 41 or 42 3018799

44 exp diet/ 399482

45 dietary pattern/4055

46 exp meal/ 24982

47 exp food/ 1216447

48 eating habit/ 13250

49 exp food intake/ 372662

50 (pattern* adj3 (diet* or eat* or meal* or food*)).tw. 25121

51 ((eat* or diet* or meal*) adj3 habit*).tw. 30390

52 (diet* adj3 (score* or pattern* or intake* or treatment* or quality)).tw. 116722

53 (meal* adj3 (score* or pattern* or intake* or treatment* or quality)).tw. 6168

54 (food* adj3 (score* or pattern* or intake* or treatment* or quality)).tw. 95890

55 (diet* adj3 (artificial or atkins or carb* or carb* loading or cariogenic or casein free or cereal base* or DASH or element* or eliminat* or experiment* or fib* free or gluten free or healthy or high calorie* or high fib* or high glycemic index or high GI or high salt or high sodium or intuitive eat* or keto* or lactose free or lipid* or liquid or low calorie* or low carb* or low fib* or low FODMAP or low glycemic index or low GI or low iodine or low residue* or macrobiotic or Mediterranean or

1

nordic or obesogen* or paleo* or protein or raw food or soft or unhealthy or vegetarian or vegan or western*)).mp. 226522

56 diet*.mp. 1136471

57 food*.mp. 983573

58 food group*.mp. 7991

59 44 or 45 or 46 or 47 or 48 or 49 or 50 or 51 or 52 or 53 or 54 or 55 or 56 or 57 or 58 2673013

60 pain/ 363994

61 chronic pain/ 66912

62 exp inflammatory pain/ 3418

63 musculoskeletal pain/ 12439

64 knee pain/ 18757

65 hip pain/ 8672

66 hand pain/ 2578

67 spinal pain/ 3678

68 rigidity/51520

69 swelling/ 56749

70 joint swelling/ 8848

71 pain*.mp. 1528430

72 ach*.mp. 1733735

73 stiff*.mp. 140132

74 tender*.mp. 62660

75 tight*.mp. 177724

76 swell*.mp. 190226

77 (symptom* adj3 (pain or stiff* or tender* or tight* or swell*)).mp. 48454

78 60 or 61 or 62 or 63 or 64 or 65 or 66 or 67 or 68 or 69 or 70 or 71 or 72 or 73 or 74 or 75 or 76 or 77 3592955

79 exp joint/ 543693

80 joint*.tw. 480666

81 79 or 80 847493

82 12 and 43 and 59 and 78 and 81 2044

EBM Reviews - Cochrane Central Register of Controlled Trials <July 2021>

1 exp aged/ 217072

2 middle aged/ 329804

3 ((More or over or greater) adj2 (45 year* or 45 yr*)).tw. 344

4 ((Adult* or population* or people* or individual* or person*) adj2 (old* or age* or elder*)).tw. 47782

5 ((Adult* or population* or people* or individual* or person*) adj2 middle age*).tw. 1413

6 age*.tw. 492523

7 elder*.tw. 49406

8 old*.tw. 121757

9 middle age*.tw.4684

10 aging/ 3759

11 1 or 2 or 3 or 4 or 5 or 6 or 7 or 8 or 9 or 10 801791

12 (symptom* adj2 disease*).tw. 5857

13 symptom*.tw. 211801

14 (symptom* adj2 osteoarthrit*).tw. 1279

15 exp Osteoarthritis/ 7977

16 exp Cartilage/ 1033

17 Menisci, Tibial/ 190

18 Bone Marrow/ 637

19 Osteophyte/ 21

20 Synovitis/ 194

21 exp Ligaments/ 1165

22 osteoarthriti*.tw. 16457

23 cartilag*.mp. 3631

24 chondral.mp. 214

25 meniscal.mp. 576

26 meniscus.mp. 1012

27 bone marrow*.mp. 15282

28 subchondral.mp. 402

29 osteophyte*.mp. 473

30 effus*.mp. 4913

31 synovitis.mp. 1200

32 ligament*.mp. 6500

33 fat pad*.mp. 308

34 attrit*.mp. 4046

35 12 or 13 or 14 or 15 or 16 or 17 or 18 or 19 or 20 or 21 or 22 or 23 or 24 or 25 or 26 or 27 or 28 or 29 or 30 or 31 or 32 or 33 or 34 253968

36 exp diet/ 18390

37 exp meals/ 1499

38 exp food/ 51411

39 exp eating/ 3740

40 (pattern* adj3 (diet* or eat* or meal* or food*)).tw. 2642

41 ((eat* or diet* or meal*) adj3 habit*).tw. 4007

42 (diet* adj3 (score* or pattern* or intake* or treatment* or quality)).tw. 17574

43 (meal* adj3 (score* or pattern* or intake* or treatment* or quality)).tw. 2469

44 (food* adj3 (score* or pattern* or intake* or treatment* or quality)).tw. 10106

45 (diet* adj3 (artificial or atkins or carb* or carb* loading or cariogenic or casein free or cereal base* or DASH or element* or eliminat* or experiment* or fib* free or gluten free or healthy or high calorie* or high fib* or high glycemic index or high GI or high salt or high sodium or intuitive eat* or keto* or lactose free or lipid* or liquid or low calorie* or low carb* or low fib* or low FODMAP or low glycemic index or low GI or low iodine or low residue* or macrobiotic or Mediterranean or nordic or obesogen* or paleo* or protein or raw food or soft or unhealthy or vegetarian or vegan or western*)).mp. 25097

46 diet*.mp. 98839

47 food*.mp. 52852

48 food group*.mp. 753

49 36 or 37 or 38 or 39 or 40 or 41 or 42 or 43 or 44 or 45 or 46 or 47 or 48 152631

50 pain/ 11821

51 exp Musculoskeletal Pain/ 1074

52 chronic pain/ 2662

53 inflammat* pain.mp. 598

54	pain*.mp.	203559
55	ach*.mp.	124029
56	stiff*.mp.	11279
57	tender*.mp.	6301
58	tight*.mp.	4391
59	swell*.mp.	8930
60	(symptom* adj3 (pain or stiff* or tender* or tight* or swell*)).mp.	10596
61	50 or 51 or 52 or 53 or 54 or 55 or 56 or 57 or 58 or 59 or 60	325125
62	exp Joints/	8184
63	joint*.tw.	32835
64	62 or 63	38062
65	11 and 35 and 49 and 61 and 64	380

Web of Science

(TS=("middle age*") OR TS=(age*) OR TS=(aging) OR TS=((Adult* or population* or people* or individual* or person*) near/2 (old* or age* or elder*)) OR TS=((Adult* or population* or people* or individual* or person*) near/2 middle age*) OR TS=((More or over or greater) near/2 (45 year*)) OR TS=(elder*) OR TS=(old*)) AND (TS=(symptom* near/2 disease*) OR TS=(symptom*) OR TS=(symptom* near/2 osteoarthrit*) OR TS=(osteoarthrit*) OR TS=(cartilag*) OR TS=(chondral) OR TS=(meniscal) OR TS=(meniscus) OR TS=("bone marrow*") OR TS=(subchondral) OR TS=(osteophyte*) OR TS=(effus*) OR TS=(synovitis) OR TS=(ligament*) OR TS=("fat pad*") OR TS=(attrit*)) AND (TS=(diet* near/3 (score* or pattern* or intake* or treatment* or quality)) OR TS=(meal* near/3 (score* or pattern* or intake* or treatment* or quality)) OR TS=(food* near/3 (score* or pattern* or intake* or treatment* or quality)) OR TS=(pattern* near/3 (diet* or eat* or meal* or food*)) OR TS=((eat* or diet* or meal*) near/3 habit*) OR TS=(diet*) OR TS=(food*) OR TS=(food group*) OR TS=(diet* near/3 (artificial or atkins or carb* or "carb* loading" or cariogenic or "casein free" or "cereal base*" or DASH or element* or eliminat* or experiment* or "fib* free" or "gluten free" or healthy or "high calorie*" or "high fib*" or "high glycemic index" or "high GI" or "high salt" or "high sodium" or "intuitive eat*" or keto* or "lactose free" or lipid* or liquid or "low calorie*" or "low carb*" or "low fib*" or "low FODMAP" or "low glycemic index" or "low GI" or "low iodine" or "low residue*" or macrobiotic or Mediterranean or nordic or obesogen* or paleo* or protein or "raw food" or soft or unhealthy or vegetarian or vegan or western*))) AND (TS=(pain*) OR TS=("Musculoskeletal Pain*") OR TS=("chronic pain*") OR TS=(ach*) OR TS=(stiff*) OR TS=(tender*) OR TS=(tight*) OR TS=(swell*) OR TS=(symptom* near/3 (pain or stiff* or tender* or tight* or swell*))) AND TS=(joint*)

Cumulative Index of Nursing and Allied Health Literature (CINAHL)

Wednesday, September 01, 2021 3:52:48 AM

#	Query	Limiters/Expanders	Last Run Via	Results
S66	S10 AND S34 AND S51 AND S64 AND S65	Expanders - Apply equivalent subjects<br>Search modes - Boolean/Phrase	Interface - EBSCOhost Research Databases<br>Search Screen - Advanced Search<br>Database - CINAHL Complete	321
S65	S54 OR S55 OR S56 OR S57 OR S58 OR S59 OR S60 OR S61 OR S62 OR S63	Expanders - Apply equivalent subjects<br>Search modes - Boolean/Phrase	Interface - EBSCOhost Research Databases<br>Search Screen - Advanced Search<br>Database - CINAHL Complete	594,748
S64	S52 OR S53	Expanders - Apply equivalent subjects<br>Search modes - Boolean/Phrase	Interface - EBSCOhost Research Databases<br>Search Screen - Advanced Search<br>Database - CINAHL Complete	160,461
S63	(MH "Pain")	Expanders - Apply equivalent subjects<br>Search modes - Boolean/Phrase	Interface - EBSCOhost Research Databases<br>Search Screen - Advanced Search<br>Database - CINAHL Complete	76,674
S62	(MH "Chronic Pain")	Expanders - Apply equivalent subjects<br>Search modes - Boolean/Phrase	Interface - EBSCOhost Research Databases<br>Search Screen - Advanced Search<br>Database - CINAHL Complete	23,927
S61	(MH "Knee Pain+")	Expanders - Apply equivalent subjects<br>Search modes - Boolean/Phrase	Interface - EBSCOhost Research Databases<br>Search Screen - Advanced Search<br>Database - CINAHL Complete	2,996
S60	pain*	Expanders - Apply equivalent subjects<br>Search modes - Boolean/Phrase	Interface - EBSCOhost Research Databases<br>Search Screen - Advanced Search<br>Database - CINAHL Complete	349,383
S59	ach*	Expanders - Apply equivalent subjects<br>Search modes - Boolean/Phrase	Interface - EBSCOhost Research Databases<br>Search Screen - Advanced	228,169

			Search<br>Database - CINAHL Complete	
S58	stiff*	Expanders - Apply equivalent subjects<br>Search modes - Boolean/Phrase	Interface - EBSCOhost Research Databases<br>Search Screen - Advanced Search<br>Database - CINAHL Complete	19,393
S57	tender*	Expanders - Apply equivalent subjects<br>Search modes - Boolean/Phrase	Interface - EBSCOhost Research Databases<br>Search Screen - Advanced Search<br>Database - CINAHL Complete	6,730
S56	tight*	Expanders - Apply equivalent subjects<br>Search modes - Boolean/Phrase	Interface - EBSCOhost Research Databases<br>Search Screen - Advanced Search<br>Database - CINAHL Complete	11,677
S55	swell*	Expanders - Apply equivalent subjects<br>Search modes - Boolean/Phrase	Interface - EBSCOhost Research Databases<br>Search Screen - Advanced Search<br>Database - CINAHL Complete	13,255
S54	symptom* N3 (pain or stiff* or tender* or tight* or swell*)	Expanders - Apply equivalent subjects<br>Search modes - Boolean/Phrase	Interface - EBSCOhost Research Databases<br>Search Screen - Advanced Search<br>Database - CINAHL Complete	15,425
S53	(MH "Joints+")	Expanders - Apply equivalent subjects<br>Search modes - Boolean/Phrase	Interface - EBSCOhost Research Databases<br>Search Screen - Advanced Search<br>Database - CINAHL Complete	70,276
S52	joint*	Expanders - Apply equivalent subjects<br>Search modes - Boolean/Phrase	Interface - EBSCOhost Research Databases<br>Search Screen - Advanced Search<br>Database - CINAHL Complete	150,337
S51	S35 OR S36 OR S37 OR S38 OR S39 OR S40 OR S41 OR S42 OR S43 OR S44 OR S45 OR S46 OR S47 OR S48 OR S49 OR S50	Expanders - Apply equivalent subjects<br>Search modes - Boolean/Phrase	Interface - EBSCOhost Research Databases<br>Search Screen - Advanced Search<br>Database - CINAHL Complete	467,733
S50	(MH "Diet+")	Expanders - Apply	Interface - EBSCOhost	129,577

		equivalent subjects<br>Search modes - Boolean/Phrase	Research Databases<br>Search Screen - Advanced Search<br>Database - CINAHL Complete	
S49	(MH "Food and Beverages+")	Expanders - Apply equivalent subjects<br>Search modes - Boolean/Phrase	Interface - EBSCOhost Research Databases<br>Search Screen - Advanced Search<br>Database - CINAHL Complete	210,648
S48	(MH "Food+")	Expanders - Apply equivalent subjects<br>Search modes - Boolean/Phrase	Interface - EBSCOhost Research Databases<br>Search Screen - Advanced Search<br>Database - CINAHL Complete	187,449
S47	(MH "Food Habits")	Expanders - Apply equivalent subjects<br>Search modes - Boolean/Phrase	Interface - EBSCOhost Research Databases<br>Search Screen - Advanced Search<br>Database - CINAHL Complete	14,721
S46	(MH "Eating")	Expanders - Apply equivalent subjects<br>Search modes - Boolean/Phrase	Interface - EBSCOhost Research Databases<br>Search Screen - Advanced Search<br>Database - CINAHL Complete	7,032
S45	(MH "Food Intake+")	Expanders - Apply equivalent subjects<br>Search modes - Boolean/Phrase	Interface - EBSCOhost Research Databases<br>Search Screen - Advanced Search<br>Database - CINAHL Complete	17,777
S44	pattern* N3 (diet* or eat* or meal* or food*)	Expanders - Apply equivalent subjects<br>Search modes - Boolean/Phrase	Interface - EBSCOhost Research Databases<br>Search Screen - Advanced Search<br>Database - CINAHL Complete	9,174
S43	(eat* or diet* or meal*) N3 habit*	Expanders - Apply equivalent subjects<br>Search modes - Boolean/Phrase	Interface - EBSCOhost Research Databases<br>Search Screen - Advanced Search<br>Database - CINAHL Complete	20,895
S42	diet* N3 (score* or pattern* or intake* or treatment* or quality)	Expanders - Apply equivalent subjects<br>Search modes - Boolean/Phrase	Interface - EBSCOhost Research Databases<br>Search Screen - Advanced Search<br>Database - CINAHL Complete	35,135

S41	meal* N3 (score* or pattern* or intake* or treatment* or quality)	Expanders - Apply equivalent subjects<br>Search modes - Boolean/Phrase	Interface - EBSCOhost Research Databases<br>Search Screen - Advanced Search<br>Database - CINAHL Complete	2,014
S40	food* N3 (score* or pattern* or intake* or treatment* or quality)	Expanders - Apply equivalent subjects<br>Search modes - Boolean/Phrase	Interface - EBSCOhost Research Databases<br>Search Screen - Advanced Search<br>Database - CINAHL Complete	32,250
S39	food* N3 (pattern* or intake* or treatment* or quality)	Expanders - Apply equivalent subjects<br>Search modes - Boolean/Phrase	Interface - EBSCOhost Research Databases<br>Search Screen - Advanced Search<br>Database - CINAHL Complete	31,647
S38	diet* N3 (artificial or atkins or carb* or "carb* loading" or cariogenic or "casein free" or "cereal base*" or DASH or element* or eliminat* or experiment* or "fib* free" or "gluten free" or healthy or "high calorie*" or "high fib*" or "high glycemic index" or "high GI" or "high salt or high sodium" or "intuitive eat*" or keto* or "lactose free" or lipid* or liquid or "low calorie*" or "low carb*" or "low fib*" or "low FODMAP" or "low glycemic index" or "low GI" or "low iodine" or "low residue*" or macrobiotic or Mediterranean or nordic or obesogen* or paleo* or protein or "raw food" or soft or unhealthy or vegetarian or vegan or western*)	Expanders - Apply equivalent subjects<br>Search modes - Boolean/Phrase	Interface - EBSCOhost Research Databases<br>Search Screen - Advanced Search<br>Database - CINAHL Complete	43,503
S37	diet*	Expanders - Apply equivalent subjects<br>Search modes - Boolean/Phrase	Interface - EBSCOhost Research Databases<br>Search Screen - Advanced Search<br>Database - CINAHL Complete	251,073

S36	food*	Expanders - Apply equivalent subjects Search modes - Boolean/Phrase	Interface - EBSCOhost Research Databases Search Screen - Advanced Search Database - CINAHL Complete	203,527
S35	"food group*"	Expanders - Apply equivalent subjects Search modes - Boolean/Phrase	Interface - EBSCOhost Research Databases Search Screen - Advanced Search Database - CINAHL Complete	2,881
S34	S11 OR S12 OR S13 OR S14 OR S15 OR S16 OR S17 OR S18 OR S19 OR S20 OR S21 OR S22 OR S23 OR S24 OR S25 OR S26 OR S27 OR S28 OR S29 OR S30 OR S31 OR S32 OR S33	Expanders - Apply equivalent subjects Search modes - Boolean/Phrase	Interface - EBSCOhost Research Databases Search Screen - Advanced Search Database - CINAHL Complete	529,182
S33	(MH "Symptoms")	Expanders - Apply equivalent subjects Search modes - Boolean/Phrase	Interface - EBSCOhost Research Databases Search Screen - Advanced Search Database - CINAHL Complete	8,451
S32	symptom* N2 disease*	Expanders - Apply equivalent subjects Search modes - Boolean/Phrase	Interface - EBSCOhost Research Databases Search Screen - Advanced Search Database - CINAHL Complete	20,594
S31	symptom*	Expanders - Apply equivalent subjects Search modes - Boolean/Phrase	Interface - EBSCOhost Research Databases Search Screen - Advanced Search Database - CINAHL Complete	410,400
S30	symptom* N2 osteoarthrit*	Expanders - Apply equivalent subjects Search modes - Boolean/Phrase	Interface - EBSCOhost Research Databases Search Screen - Advanced Search Database - CINAHL Complete	1,534
S29	(MH "Osteoarthritis+")	Expanders - Apply equivalent subjects Search modes - Boolean/Phrase	Interface - EBSCOhost Research Databases Search Screen - Advanced Search Database - CINAHL Complete	30,630
S28	(MH "Cartilage+")	Expanders - Apply equivalent subjects	Interface - EBSCOhost Research Databases	18,806

		Search modes - Boolean/Phrase	Search Screen - Advanced Search<br>Database - CINAHL Complete	
S27	(MH "Menisci, Tibial")	Expanders - Apply equivalent subjects<br>Search modes - Boolean/Phrase	Interface - EBSCOhost Research Databases<br>Search Screen - Advanced Search<br>Database - CINAHL Complete	2,684
S26	(MH "Bone Marrow")	Expanders - Apply equivalent subjects<br>Search modes - Boolean/Phrase	Interface - EBSCOhost Research Databases<br>Search Screen - Advanced Search<br>Database - CINAHL Complete	6,222
S25	(MH "Synovitis+")	Expanders - Apply equivalent subjects<br>Search modes - Boolean/Phrase	Interface - EBSCOhost Research Databases<br>Search Screen - Advanced Search<br>Database - CINAHL Complete	1,814
S24	(MH "Ligaments+")	Expanders - Apply equivalent subjects<br>Search modes - Boolean/Phrase	Interface - EBSCOhost Research Databases<br>Search Screen - Advanced Search<br>Database - CINAHL Complete	11,707
S23	osteoarthrit*	Expanders - Apply equivalent subjects<br>Search modes - Boolean/Phrase	Interface - EBSCOhost Research Databases<br>Search Screen - Advanced Search<br>Database - CINAHL Complete	40,882
S22	cartilag*	Expanders - Apply equivalent subjects<br>Search modes - Boolean/Phrase	Interface - EBSCOhost Research Databases<br>Search Screen - Advanced Search<br>Database - CINAHL Complete	20,432
S21	chondral	Expanders - Apply equivalent subjects<br>Search modes - Boolean/Phrase	Interface - EBSCOhost Research Databases<br>Search Screen - Advanced Search<br>Database - CINAHL Complete	1,652
S20	meniscal	Expanders - Apply equivalent subjects<br>Search modes - Boolean/Phrase	Interface - EBSCOhost Research Databases<br>Search Screen - Advanced Search<br>Database - CINAHL Complete	4,023
S19	meniscus	Expanders - Apply	Interface - EBSCOhost	4,032

		equivalent subjects<br>Search modes - Boolean/Phrase	Research Databases<br>Search Screen - Advanced Search<br>Database - CINAHL Complete	
S18	"bone marrow*"	Expanders - Apply equivalent subjects<br>Search modes - Boolean/Phrase	Interface - EBSCOhost Research Databases<br>Search Screen - Advanced Search<br>Database - CINAHL Complete	23,368
S17	subchondral	Expanders - Apply equivalent subjects<br>Search modes - Boolean/Phrase	Interface - EBSCOhost Research Databases<br>Search Screen - Advanced Search<br>Database - CINAHL Complete	2,234
S16	osteophyte*	Expanders - Apply equivalent subjects<br>Search modes - Boolean/Phrase	Interface - EBSCOhost Research Databases<br>Search Screen - Advanced Search<br>Database - CINAHL Complete	1,705
S15	effus*	Expanders - Apply equivalent subjects<br>Search modes - Boolean/Phrase	Interface - EBSCOhost Research Databases<br>Search Screen - Advanced Search<br>Database - CINAHL Complete	12,908
S14	synovitis	Expanders - Apply equivalent subjects<br>Search modes - Boolean/Phrase	Interface - EBSCOhost Research Databases<br>Search Screen - Advanced Search<br>Database - CINAHL Complete	3,055
S13	ligament*	Expanders - Apply equivalent subjects<br>Search modes - Boolean/Phrase	Interface - EBSCOhost Research Databases<br>Search Screen - Advanced Search<br>Database - CINAHL Complete	31,798
S12	"fat pad*"	Expanders - Apply equivalent subjects<br>Search modes - Boolean/Phrase	Interface - EBSCOhost Research Databases<br>Search Screen - Advanced Search<br>Database - CINAHL Complete	1,117
S11	attrit*	Expanders - Apply equivalent subjects<br>Search modes - Boolean/Phrase	Interface - EBSCOhost Research Databases<br>Search Screen - Advanced Search<br>Database - CINAHL Complete	5,625

S10	S1 OR S2 OR S3 OR S4 OR S5 OR S6 OR S7 OR S8 OR S9	Expanders - Apply equivalent subjects<br>Search modes - Boolean/Phrase	Interface - EBSCOhost Research Databases<br>Search Screen - Advanced Search<br>Database - CINAHL Complete	2,315,007
S9	old*	Expanders - Apply equivalent subjects<br>Search modes - Boolean/Phrase	Interface - EBSCOhost Research Databases<br>Search Screen - Advanced Search<br>Database - CINAHL Complete	374,263
S8	elder*	Expanders - Apply equivalent subjects<br>Search modes - Boolean/Phrase	Interface - EBSCOhost Research Databases<br>Search Screen - Advanced Search<br>Database - CINAHL Complete	118,603
S7	age*	Expanders - Apply equivalent subjects<br>Search modes - Boolean/Phrase	Interface - EBSCOhost Research Databases<br>Search Screen - Advanced Search<br>Database - CINAHL Complete	2,223,767
S6	(Adult* or population* or people* or individual* or person*) N2 "middle age*"	Expanders - Apply equivalent subjects<br>Search modes - Boolean/Phrase	Interface - EBSCOhost Research Databases<br>Search Screen - Advanced Search<br>Database - CINAHL Complete	792,440
S5	(Adult* or population* or people* or individual* or person*) N2 (old* or age* or elder*)	Expanders - Apply equivalent subjects<br>Search modes - Boolean/Phrase	Interface - EBSCOhost Research Databases<br>Search Screen - Advanced Search<br>Database - CINAHL Complete	1,315,399
S4	(More or over or greater) N2 (45 year* or 45 yr*)	Expanders - Apply equivalent subjects<br>Search modes - Boolean/Phrase	Interface - EBSCOhost Research Databases<br>Search Screen - Advanced Search<br>Database - CINAHL Complete	659
S3	"middle age*"	Expanders - Apply equivalent subjects<br>Search modes - Boolean/Phrase	Interface - EBSCOhost Research Databases<br>Search Screen - Advanced Search<br>Database - CINAHL Complete	1,076,592
S2	(MH "Aged+")	Expanders - Apply equivalent subjects<br>Search modes - Boolean/Phrase	Interface - EBSCOhost Research Databases<br>Search Screen - Advanced Search<br>Database - CINAHL Complete	885,567
